# Cooperative DNA binding mediated by KicGAS/ORF52 oligomerization allows inhibition of DNA-induced phase separation and activation of cGAS

**DOI:** 10.1093/nar/gkab689

**Published:** 2021-08-13

**Authors:** Debipreeta Bhowmik, Mingjian Du, Yuan Tian, Siming Ma, Jianjun Wu, Zhijian Chen, Qian Yin, Fanxiu Zhu

**Affiliations:** Department of Biological Science, Florida State University, Tallahassee, FL 32306, USA; Department of Molecular Biology, University of Texas Southwestern Medical Center, Dallas, TX75390-9148, USA; Department of Biological Science, Florida State University, Tallahassee, FL 32306, USA; Department of Biological Science, Florida State University, Tallahassee, FL 32306, USA; Department of Biological Science, Florida State University, Tallahassee, FL 32306, USA; Department of Molecular Biology, University of Texas Southwestern Medical Center, Dallas, TX75390-9148, USA; Howard Hughes Medical Institute, 4000 Jones Bridge Rd, Chevy Chase, MD 20815, USA; Department of Biological Science, Florida State University, Tallahassee, FL 32306, USA; Department of Biological Science, Florida State University, Tallahassee, FL 32306, USA

## Abstract

Cyclic GMP-AMP synthase (cGAS) is a key DNA sensor that detects aberrant cytosolic DNA arising from pathogen invasions or genotoxic stresses. Upon binding to DNA, cGAS is activated and catalyzes the synthesis of cyclic GMP-AMP (cGAMP), which induces potent antimicrobial and antitumor responses. Kaposi sarcoma-associated herpesvirus (KSHV) is a human DNA tumor virus that causes Kaposi sarcoma and several other malignancies. We previously reported that **K**SHV **i**nhibitor of **cGAS** (KicGAS) encoded by ORF52, inhibits cGAS enzymatic activity, but the underlying mechanisms remained unclear. To define the inhibitory mechanisms, here we performed in-depth biochemical and functional characterizations of KicGAS, and mapped its functional domains. We found KicGAS self-oligomerizes and binds to double stranded DNA cooperatively. This self-oligomerization is essential for its DNA binding and cGAS inhibition. Interestingly, KicGAS forms liquid droplets upon binding to DNA, which requires collective multivalent interactions with DNA mediated by both structured and disordered domains coordinated through the self-oligomerization of KicGAS. We also observed that KicGAS inhibits the DNA-induced phase separation and activation of cGAS. Our findings reveal a novel mechanism by which DNA viruses target the host protein phase separation for suppression of the host sensing of viral nucleic acids.

## Graphical Abstract

**Graphical Abstract:**
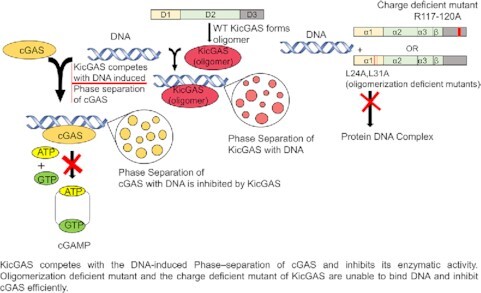
KicGAS competes with the DNA-induced phase–separation of cGAS and inhibits its enzymatic activity. Oligomerization deficient mutant and the charge deficient mutant of KicGAS are unable to bind DNA and inhibit cGAS efficiently.

## INTRODUCTION

Aberrant DNAs can be sensed by innate immune sensors to alarm the host of microbial pathogens as well as damaged or malignant cells. One of such sensors is cyclic GMP-AMP synthase (cGAS) (1). Upon binding to dsDNA, cGAS dimerizes, assembles into liquid droplets, and is activated to catalyze the synthesis of cyclic GMP-AMP (cGAMP) (2–6). As a secondary messenger, cGAMP activates endoplasmic reticulum-bound adaptor protein STING (STimulator of Interferon Genes) in the same cell or neighboring cells. Activated STING recruits kinase TBK1 and activates transcription factor IRF3 to initiate a downstream signaling cascade that culminates the expression of type I interferons (IFNs) and other proinflammatory cytokines (7). The cGAS–cGAMP–STING pathway exhibits broad antiviral activity and is crucial for the host defense against various DNA viruses such as herpesviruses as well as retroviruses (8–10). Besides foreign DNAs, cGAS can also sense self DNAs of damaged or malignant cells (11–13). Accumulating evidence suggests that the cGAS–cGAMP–STING pathway plays a critical role in antitumor immunity, underlying the mechanisms of common regimes of chemo- and radio- therapies that cause genotoxic stresses (14).

Kaposi's sarcoma-associated herpesvirus (KSHV) is a large DNA tumor virus that causes Kaposi sarcoma (KS), primary effusion lymphoma (PEL), and multicentric Castleman's disease (MCD) (15–17). KS was rare in general population but became prevalent among AIDS patients. There is no cure for KS, although doxorubicin, paclitaxel, and interferon have been approved by FDA for the treatment of KS with limited success (18). Introduction of highly active antiretroviral treatment (HAART) has reduced the incidence but KS remains the most common cancer among HIV-infected patients and the most common cancer in sub-Saharan Africa (19). The outcome and pathogenesis of KSHV infection depend on the interplays between the virus and the host immune responses. Because the cGAS–cGAMP–STING pathway is crucial not only for antiviral but also for antitumor immunity, KSHV is expected to encode mechanisms to evade the cGAS-DNA sensing. Indeed, we and the others have shown that several KSHV ORFs, most of them encode virion contained proteins, inhibit cGAS–cGAMP–STING signaling at various steps (20–22). In particular, we found that ORF52 or KicGAS (KSHV inhibitor of cGAS), an abundant virion component protein inhibits cGAS enzymatic activity (21). To our knowledge, KicGAS is the first reported viral inhibitor of cGAS. Although the inhibitory effect of KicGAS has been further confirmed by others (23,24), the detailed molecular mechanisms by which KicGAS inhibits cGAS remained elusive. To understand the mechanism of inhibition and to develop KicGAS as a potential therapeutic target, we performed in-depth biochemical and functional characterizations of KicGAS. KicGAS consists of three putative domains: the structured domains I and II, and the disordered domain III. We first revealed that all three putative domains of KicGAS are required for the inhibition of cGAS. In addition, KicGAS self-oligomerizes via the N-terminal domain and the oligomerization is required for efficient DNA binding and cGAS inhibition. We found that KicGAS binds to dsDNA cooperatively and in a DNA length dependent but sequence independent manners. More importantly, DNA binding to KicGAS results in the formation of liquid condensates through phase separation. Recently, it has been shown that liquid-liquid phase separation is responsible for organization of many complex biochemical reactions (25). Multivalent interactions of cGAS with DNA also promote liquid phase separation that facilitates cGAS activation and cGAMP synthesis as well as protects DNA from degradation by TREX1 nuclease in cells(2,5,26). Finally, we have shown that KicGAS interferes with the DNA-induced phase separation of cGAS and inhibits cGAS-DNA sensing. Altogether, we present a novel mechanism by which DNA viruses target the host protein phase separation for suppression of the host sensing of viral nucleic acids.

## MATERIALS AND METHODS

### Reagents

All DNA and RNA oligonucleotides were purchased from Integrated DNA Technologies (IDT) (nucleic acid sequences are shown in Table S1). Fluorescent ATP was purchased from Jena Biosciences. GTP was purchased from Invitrogen. All the other chemicals were purchased from Thermo Fisher Scientific or Sigma-Aldrich.

### Protein purification

KicGAS and its mutants were cloned into modified pET28a vector with an N-terminal Avi-His_6_-SUMO tag that was gifted from Dr Pingwei Li (27). All proteins were produced in *E.coli* Rosetta. Bacteria were grown until an OD_600_ of 0.6–0.8 and expression was induced at 20°C for 16 to 18 h with 400 μM IPTG. Recombinant KicGAS proteins were purified by successive Ni-NTA affinity, Heparin ion exchange, and Superdex 200 chromatography steps. Cells were lysed by sonication in 20 mM phosphate buffer (pH 7.4), 300 mM NaCl, 10% glycerol, 30 mM imidazole and 1 mM DTT. Clarified lysate was bound to Ni-NTA agarose (QIAGEN), and resin was washed with lysis buffer supplemented with 1M NaCl prior to the elution. The bound protein was eluted using lysis buffer supplemented with 300 mM imidazole. The purified proteins were cleaved with SUMO protease ULP1 (1:1000, w:w) at 4°C overnight. The SUMO tag was removed using a Ni-NTA column, and the target proteins in the flow through were further purified by heparin column with the use of a linear gradient of 300–1000 mM NaCl. Protein was further purified by size-exclusion chromatography on a Superdex 200 16/600 column with a buffer containing 20 mM Tris–HCl at pH 7.4, 150 mM NaCl, and 5 mM DTT. Tag-free, purified KicGAS protein was then concentrated to 10–20 mg/ml using Amicon^®^ Ultra Centrifugal Filters, frozen and stored in −80°C. Same methodology was followed for the purification of mutant proteins. Human cGAS was purified as described previously (21).

We engineered the aforementioned modified pET28a vector to express His_6_-SUMO tagged KicGAS (FL:1–131), ΔD1 (41-131), ΔD2 (1–40^108–131), ΔD3(1–107) and 9–121 KicGAS. The engineered pET28a plasmid containing the human cGAS gene was already stocked in our laboratory. All mutants were generated by QuikChange site-directed mutagenesis (Agilent) following the manufacturer's instruction and were confirmed by sequencing.

### Electrophoretic mobility shift assay

Electrophoretic mobility shift assay experiments were carried out in buffer A (20 mM Tris–HCl pH 7.4, 150 mM NaCl and 10% glycerol). To a fixed amount of FAM-ISD45 and ISD25 (20 nM), increasing concentration of KicGAS was added. The reaction was allowed to equilibrate on ice (at least 20 min), then applied to a 5% 37.5:1 acrylamide:bis-acrylamide Tris–acetic acid–EDTA gel. The gel was run at 100 V in 1× Tris-acetic acid-EDTA buffer in cold room (4°C) and imaged using a BIO-RAD ChemiDoc MP Imaging System. The gel image was analyzed using ImageJ. The data points were fitted to Hill equation using Origin 8.5 to calculate the binding affinity.

### Fluorescence polarization assay

Fluorescence polarization was measured in multimode micro-plate reader from Biotek Instruments using 384-well black non-binding PS plates (Greiner Bio-One). Increasing concentrations of KicGAS and its mutants were added to a fixed concentration of FAM-DNA (400 pg/μl). Changes in fluorescence polarization were plotted as a function of protein concentration and fitted to the Hill equation using Origin 8.5.

### cGAS inhibition assay

We have performed the cGAS activity assay (28) in the presence of purified KicGAS or its mutant proteins. A fluorescent analogue of ATP (2-aminopurine riboside-5′-*O*-triphosphate (fATP)) was used. Briefly, 400 nM ISD45 was premixed with KicGAS or its mutant in different concentrations after which 400 nM cGAS in 40 mM Tris pH 7.4, 100 mM NaCl and 200 μM Zn^2+^ was added. The reaction was started by adding 5 mM MgCl_2_ with 500 μM GTP and 50 μM fATP. Fluorescence intensity was measured in 384-well black non-binding PS plates (Greiner Bio-One) on SpectraMax iD5 (*λ*_ex_ = 307 nm, *λ*_em_ = 370 nm, kinetic interval 2 min). A negative control containing all the reaction components except the enzyme was included every time and the data of the negative control was subtracted from all the other reaction data before final processing.

### Turbidity assay

Purified proteins of wild-type KicGAS or variants and human cGAS were diluted to 10 μM in the buffer (20 mM Tris, pH 7.4 with 100, 200, 300, 400 or 500 mM NaCl). Phase separation was initiated by addition of 10 μM ISD100 to a 40 μl final volume in a Greiner 384 well transparent microplate and turbidity was measured after incubation for 0.5 h at room temperature by absorbance of 350 nm wavelength using a microplate reader SpectraMax iD5. The absorbance values were plotted against NaCl concentrations using Origin 8.5. Data are represented as means ± SD of three independent experiments.

### DNA condensation assay

Recombinant KicGAS proteins and Cy3 labeled ISD100 DNA were mixed and incubated at room temperature for 1 h in buffer A (20 mM HEPES, pH 7.4, 150 mM NaCl). Each reaction contained 10 nM Cy3-ISD100. KicGAS proteins were added from 10 nM to 1 μM with 2-fold increase for a final sample volume of 25 μl. Similarly, we used cGAS protein in the same conditions as a control. After 1 h incubation, mixtures were centrifuged at 13 000 g for 15 min (29). After separating the supernatant, the pellet was resuspended in 1 M NaCl to starting volume (30). 20 μl of supernatant and 20 μl of the redissovled pellet were transferred to a Greiner 384 wells plate. The Cy3 fluorescence intensity of each sample was quantified on the SpectraMax iD5 plate reader using 540 nm excitation and 580 nm emission wavelengths. The fluorescence intensity values of Cy3-DNA in the supernatant and the redissovled pellet were plotted against protein concentrations using Origin 8.5. Data are represented as means ± SD of three independent experiments. The input, supernatant, and redissolved pellet samples were analyzed by western blot to examine partition of protein in each fractions.

### *In vitro* phase separation assay

In general, recombinant KicGAS protein (30 μM) (3% Alexa Fluor 647-labeled) was mixed with DNA (30 μM) of a defined length (2% Cy3-labeled) in a buffer with 20 mM Tris–HCl, pH 7.5, 300 mM NaCl and in 96-well plates (Corning) coated with 20 mg/ml BSA (Sigma). Mixtures were incubated and images were captured after 60 min of incubation. For the assays of the inhibition of cGAS phase separation by KicGAS and its mutants, phase separation of recombinant cGAS (10 μM, 3% Alexa Fluor 488-labeled) with 45-bp ISD (10 μM, 2% Cy3-labeled) was performed in 20 mM Tris–HCl, pH 7.5, 150 mM NaCl in presence of 10 μM KicGAS or its mutants. Before the phase separation assay, all the proteins were dialyzed in the same buffer (20 mM Tris–HCl, pH 7.5, 150 mM NaCl).

### Image acquisition and analysis

Phase separated droplets in Figure 6, S7–9 were imaged by using Nikon A1R + confocal microscope with Nikon A1 camera, and X-Cite 120LED laser. Phase separated droplets in Figures 7 and 8 were imaged by using LSM 980 Airyscan 2 microscope (ZEISS) with a Plan-Apochromat 40×/1.2 objective, GaAsP-PMT and multialkali-PMT detectors, and a X-Cite Xylis laser. Imaging power was 1% and images were analyzed using ImageJ (NIH). Fluorescence intensities of phase-separated droplets were quantified using ImageJ (NIH).

### *In vitro* FRAP assays

FRAP experiments were performed on a Nikon A1R + confocal microscope at 25°C. For FRAP of KicGAS or DNA, spots of ∼2-μm diameter in ∼10-μm droplets were photobleached with 10% laser power for 1 s using 561- and 647-nm lasers. Time-lapse images were acquired over a 10-min time course after bleaching with 20-second interval. Images were processed by ImageJ. Fluorescence intensities of regions of interest (ROIs) were corrected by unbleached control regions and then normalized to pre-bleached intensities of the ROIs. The corrected and normalized data were fit to the single exponential model by GraphPad Prism 8: ${I_t}\, = \,{I_0}\, + \,({I_\infty } - {I_0})\,(1 - {e^{ - kt}})$ where ${I_0}$ was the intensity at the start of recovery after bleaching, ${I_\infty }$ was the plateau intensity, and $k$ was the exponential constant. The recovery rate was calculated by ${I_\infty }$ divided by the fluorescence intensity before bleaching.

## RESULTS

### KicGAS and its homologues efficiently inhibit cGAS enzymatic activity

**Figure 1.**
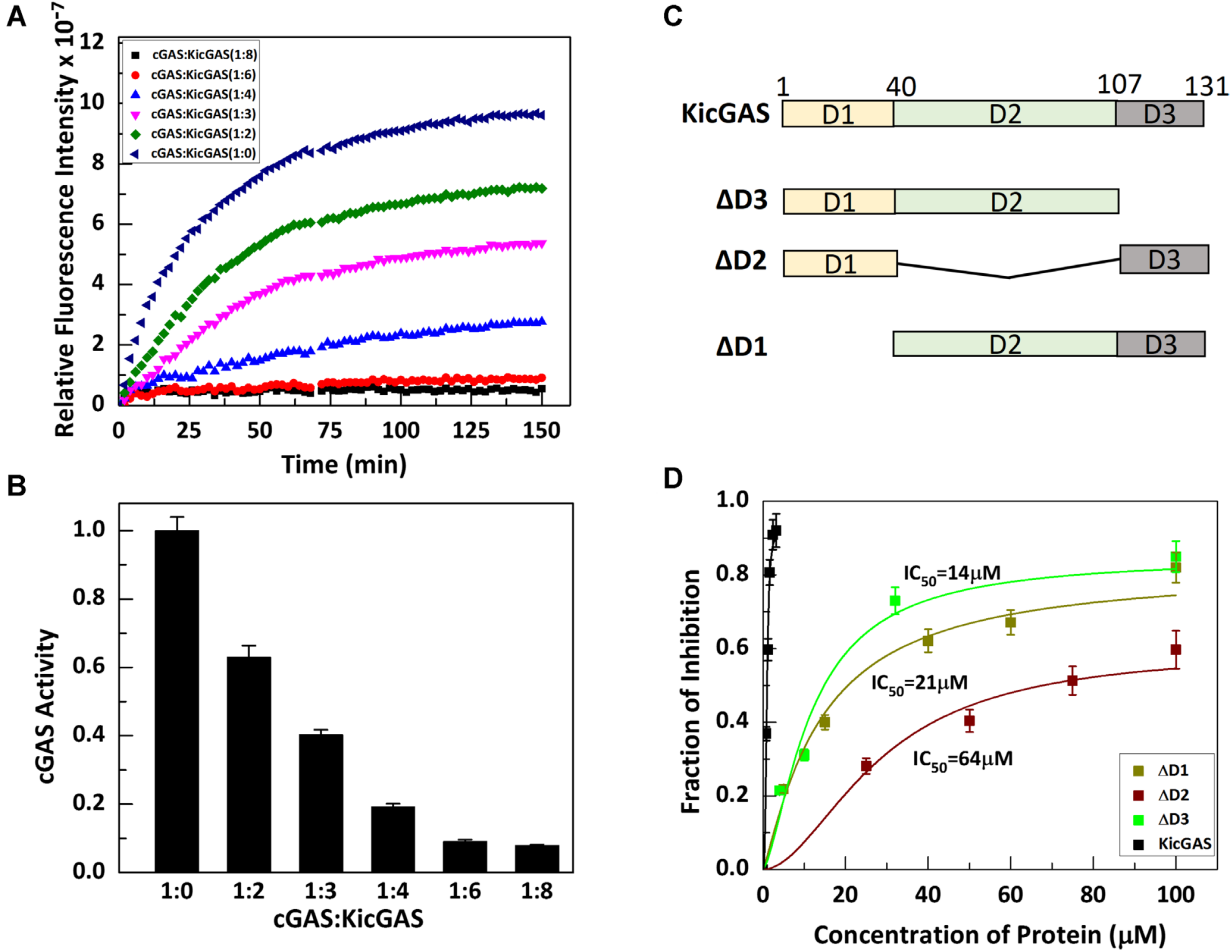
KicGAS requires all the three domains for efficiently inhibiting cGAS. (A, B) Full length protein efficiently inhibits cGAS in a dose dependent manner. (**A**) cGAS activity assay was performed in absence and presence of different concentrations of KicGAS. (**B**) Fraction of cGAS enzymatic activity retained in presence of different concentrations of KicGAS. (C, D) Deletion of any domain results reduction in cGAS inhibition. (**C**) Diagram of the domain truncated constructs of KicGAS. (**D**) Plot of the fraction of inhibition of cGAS activity in presence different concentration of truncated constructs as inhibitor.

To quantify the inhibition of cGAS enzymatic activity by KicGAS, we sought assays that can monitor the dynamics of cGAS enzymatic reaction and measure the enzymatic activity more quantitatively. We adopted an *in vitro* fluorescence based cGAS enzyme assay recently reported by Andreeva *et al.* (28). cGAS catalyzes the conversion of fluorescent ATP analogue (fATP: 2-aminopurine riboside triphosphate) into less fluorescent fcGAMP (fluorescent cGAS product), resulting in a gradual decrease in fluorescence intensity during the reaction. By monitoring the fluorescence intensity over time, we can measure the enzymatic activity of cGAS. We optimized the reaction condition and included Zn^2+^ in the reaction as it was shown to enhance the cGAS enzymatic activity (2). When increasing amounts of KicGAS were included in the reaction, we observed that KicGAS inhibited cGAS enzymatic activity in a dose dependent manner (Figure 1A and B). We estimated the IC_50_ value to be ∼0.9 μM when cGAS concentration was 0.4 μM. Indeed, purified homologues of KicGAS proteins from Rhesus macaque rhadinovirus (RRV), Murine herpesvirus 68 (MHV68), and Epstein-Barr virus (EBV) homologues also inhibited the cGAS enzymatic activity in a similar fashion (Supplementary Figure S1A). The calculated values of IC_50_ are depicted in Supplementary Figure S1A. The results confirmed that the inhibition of cGAS by KicGAS is evolutionarily conserved among γ herpesviruses.

### Near full length of KicGAS is required for cGAS inhibition

Because cGAS inhibition is conserved among KicGAS homologues, we performed multiple sequence alignment of all known homologues (Supplementary Figure S2). The analyses revealed three distinct domains, the N-terminal domain I (D1), the middle domain II (D2), and the less conserved C-terminal domain III (D3). The very ends of N- and C- termini are less conserved. We next assessed the roles of each domain in the inhibition of cGAS activity. We generated truncated proteins in which each of the three domains were deleted and found that the deletion of each domain reduced the inhibition (Figure 1C and D). The IC_50_ values for ΔD1, ΔD2, ΔD3 became 21, 64 and 14 μM respectively, in comparison to IC_50_ of 0.9 μM of the full length KicGAS protein, an increase of 23-, 70- and 15-fold, respectively (Figure 1D), suggesting each domain is required for the optimal inhibition of cGAS. We observed that deletion of the first 8 aa from the N-terminus and 10 aa from the C-terminus had little effect on the inhibition (Supplementary Figure S 1B). Together, these results suggest that near full length protein (9–121) and structural integrity of KicGAS are required for its inhibition of cGAS enzymatic activity.

### KicGAS efficiently binds to dsDNA in a length dependent but sequence independent manner

**Figure 2.**
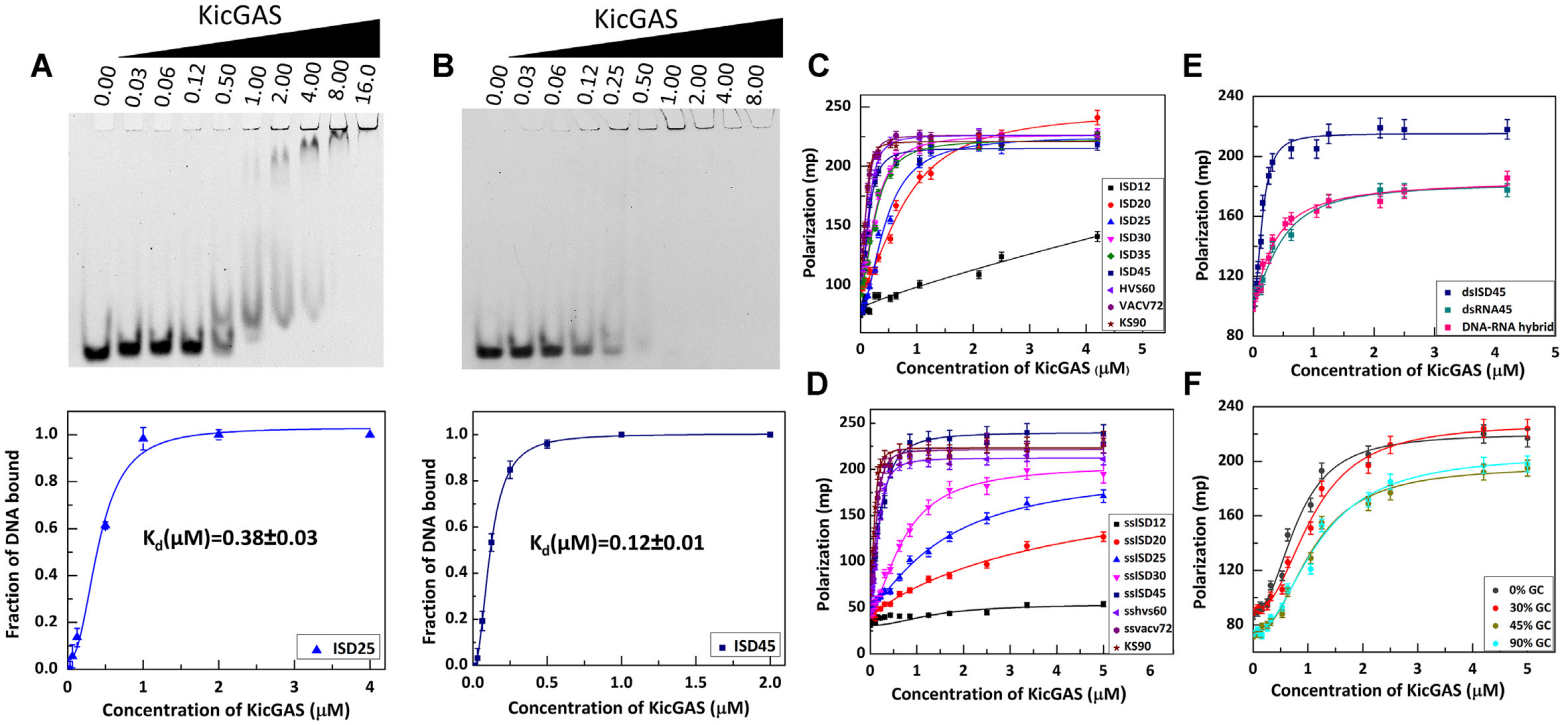
Characterization of the DNA binding of KicGAS. (A, B) *KicGAS binds efficiently to dsDNA*. Electrophoretic mobility shift assay for the binding of (**A**) ISD25 and (**B**) ISD45 with KicGAS (concentration of KicGAS is shown in μM). The EMSA results in the top panel were fitted to the Hill equation to obtain dissociation constant (*K*_d_) and the Hill coefficient (*n*). The fitted curves were shown in the bottom panel. (C–F) KicGAS shows specificity towards dsDNA and the binding is DNA length dependent but sequence independent. Binding curves of FL KicGAS with (**C**) different length of double stranded DNA, (**D**) different length of single stranded DNA, (**E**) RNA and DNA–RNA hybrid, (**F**) DNA containing different percentage of GC.

We set out further to unravel the mechanism of inhibition of cGAS by KicGAS. KicGAS is a positively charged protein and shows strong affinity towards dsDNA. We next wanted to characterize the DNA binding of KicGAS. We performed electrophoretic mobility shift assay (EMSA) (Figure 2A and B) using fluorescein amidite labelled double stranded DNA (FAM-dsDNA) and KicGAS. We observed the protein–DNA complex in the gel for shorter DNA of 25 bp (ISD25). When length of the DNA is increased to 45 bps (ISD45), although the DNA probe signal was reduced as the concentration of KicGAS increased, no shifted protein–DNA complex was easily discernable presumably due to the increase of the size of protein–DNA complex that failed to migrate into gel. Based on the depletion of free DNA probe signal, we were able to estimate the *K*_d_ of KicGAS binding to ISD25 and ISD45 and the values are 0.38 and 0.12 μM respectively (Table 1).

**Table 1.** DNA binding affinity of FL KicGAS as determined from EMSA

	ISD45	ISD25
*K*_d_(μM)	0.12 ± 0.01	0.38 ± 0.03
n	2.20 ± 0.12	1.95 ± 0.30

cGAS interacts with DNA in a length dependent manner and the interaction was found to be cooperative (28). We next wanted to determine whether DNA length affects the dsDNA binding affinity of KicGAS. We used fluorescence polarization assays to measure KicGAS binding to FAM-dsDNA of lengths ranging from 12 to 90 bp. We observed no significant binding between FAM-dsISD12 and KicGAS, but when the length was increased to 20 bp, we observed measurable binding. Both binding affinity and Hill coefficient (n) increased with the increase of DNA length (Figure 2C, Table 2), suggesting that KicGAS cooperatively binds with DNA in a length dependent manner. It is evident from the binding affinity values that about 45 base pairs are required to achieve the optimal efficiency.

**Table 2.** Binding parameters for the interaction of FL KicGAS with dsDNA of different length

	ISD12	ISD20	ISD25	ISD30	ISD35	ISD45	HSV60	VACV72	KS90
*K*_d_(μM)	nd	0.82 ± 0.08	0.44 ± 0.03	0.29 ± 0.02	0.27 ± 0.01	0.15 ± 0.02	0.16 ± 0.01	0.13 ± 0.01	0.10 ± 0.01
n	nd	1.70 ± 0.19	1.84 ± 0.23	1.88 ± 0.27	1.90 ± 0.24	2.1 ± 0.32	2.24 ± 0.24	2.36 ± 0.40	2.55 ± 0.32

KicGAS also binds to ssDNA (Figure 2D, Table 3), and the binding affinity as well as cooperativity increases with the increase in length. The binding affinity is higher for dsDNA compared to the same length of ssDNA, the difference is more prominent for shorter length of DNA (12–30 bp) where the binding curves for ssDNA are more hyperbolic, suggesting that the lower cooperativity results in weaker affinity. KicGAS appears to bind to both dsRNA and DNA–RNA hybrid (Figure 2E, Table 4), although the affinity is lower than dsDNA of the same length. It is to be noted that cGAS can discriminate dsDNA, dsRNA, DNA–RNA hybrid in a similar fashion (31) although the discrimination appears much clearer in case of cGAS compared to KicGAS.

**Table 3.** Binding parameters for the interaction of FL KicGAS with ssDNA of different length

	ssISD12	ssISD20	ssISD25	ssISD30	ssISD45	ssHSV60	ssVACV72	ssKS90
*K*_d_(μM)	nd	5.08 ± 0.40	1.58 ± 0.22	0.72 ± 0.05	0.26 ± 0.01	0.17 ± 0.01	0.13 ± 0.01	0.10 ± 0.01
n	nd	0.90 ± 0.23	1.33 ± 0.16	1.50 ± 0.15	1.85 ± 0.17	2.15 ± 0.20	2.21 ± 0.39	2.24 ± 0.41

**Table 4.** Binding parameters for the interaction of FL KicGAS with dsDNA, dsRNA, DNA–RNA hybrid

	ISD45	RNA45	DNA–RNA hybrid
*K*_d_ (μM)	0.15 ± 0.02	0.56 ± 0.05	0.30 ± 0.02
n	2.1 ± 0.32	1.69 ± 0.27	1.85 ± 0.22

The next question is whether the sequence of dsDNAs affects binding to KicGAS. We used DNAs of varying GC content but of the same length in the fluorescence polarization assay (Figure 2F, Table 5). We obtained almost similar binding affinity curve suggesting that binding is sequence independent. In summary, KicGAS binds to DNA cooperatively in a length dependent but sequence independent manner. KicGAS appears to be able to distinguish dsDNA from ssDNA or RNAs with short length but the difference diminished when the length of nucleic acids increases.

**Table 5.** Binding parameters for the interaction of FL KicGAS with DNA having different GC content

	0%	30%	45%	90%
*K*_d_ (μM)	0.80 ± 0.05	1.07 ± 0.06	1.08 ± 0.06	1.16 ± 0.08
n	2.33 ± 0.33	2.31 ± 0.28	2.25 ± 0.30	2.05 ± 0.29

### Domain I mediated oligomerization of KicGAS is crucial for cooperative DNA binding and cGAS inhibition

Having determined the properties of full length KicGAS binding to DNA, we next examined the truncated proteins ΔD1, ΔD2 and ΔD3 to elucidate the role of each domain in DNA binding. FP assays revealed that deletion of any domain abolished DNA binding (Supplementary Figure S3A) suggesting that all three domains collectively contribute to binding to DNA and that the loss of DNA binding correlates well with the loss of cGAS inhibition (Figures 1D and S3A). Among three domains, both domain I and domain II are more conserved and structured, but domain III is less conserved and found to be disordered (Supplementary Figure S2).

**Figure 3.**
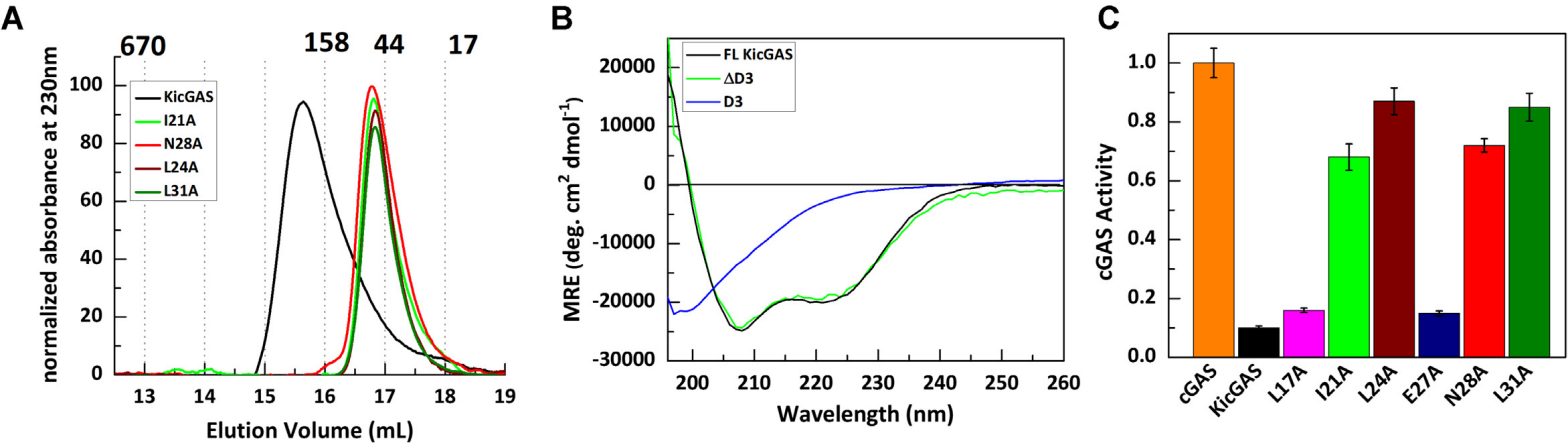
Identification of the conserved residues in domain I, that are crucial for KicGAS oligomerization and cGAS inhibition. (**A**) Size exclusion chromatograph (Superose 6 increase) of the FL KicGAS and domain I mutants I21A, L24A, N28A and L31A (**B**) The domain III is disordered. Circular dichroic spectra of FL KicGAS, ΔD3 and D3. (**C**) Bar graph showing the fraction of cGAS activity retained in presence of domain I mutants (at cGAS:KicGAS/mutant = 1:6 molar ratio).

First, we wanted to identify the structural elements and features in domain I and II that contribute to cGAS inhibition. We noticed that KicGAS is capable of self oligomerization, evident from size exclusion chromatogram (Figure 3A). Because deletion of domain I abolished the oligomeization (Supplementary Figure S3B), while deletion of domain III has little effect, we reasoned that the domain I is critical for KicGAS self-oligomerization. The multiple sequence alignments revealed four invariably conserved residues (L24, E27, N28 and L31) and two highly conserved residues (L17 and I21) located in domain I. We therefore mutated all these conserved residues individually to alanines. We found that I21A, L24A, N28A and L31A mutations resulted in shift of the size exclusion chromatographs towards low molecular weight (Figure 3A), suggesting critical roles of these residues in mediating oligomerization. We have also performed circular dichroic study to ensure that these mutations does not cause any structural change in this domain (Supplementary Figure S3C). In contrast, L17A and E27A showed no change in oligomeric behavior.

We next asked whether oligomerization affects cGAS inhibition and DNA binding. We found that each of the oligomerization deficient mutants had much reduced DNA binding and cGAS inhibition (Supplementary Figure S3D and C, Table 7). I21, L24, N28, L31 appeared the most crucial because change of these residues to alanines resulted in 10–20-fold increase of *K*_d_. The reduction of DNA binding correlated well with the reduction of cGAS inhibition (Figure 3C), L24A and L31A showed highest reduction in DNA binding and lost cGAS inhibition, I21A and N28A had intermediate reduction in DNA binding and cGAS inhibition, while L17A and E27A mutants (which didn’t affect oligomerization) behaved almost similarly as the wild type protein. These results suggest the oligomerization is mediated by domain I through the conserved residues and these residues are important for DNA binding and thus cGAS inhibition.

### Strictly conserved R97 in domain II is important for DNA binding and inhibition of cGAS

Because deletion of domain II had the most dramatic effect on cGAS inhibition (Figure 1D), we next wanted to characterize structural elements and feature in this domain that contribute to cGAS inhibition. Domain II is not only conserved among γ herpesviruses but also similar to a structural fold found in VP22 of herpes simplex virus (32), an α herpesvirus, we therefore focused on those residues that are conserved within γ herpesvirus, and between γ and α herpesviruses. We mutated the remaining strictly conserved R97 and several other highly conserved residues L55, L62, I70, V74, V87, L91, L96, I98 and V100 (Supplementary Figure S2 and Table S2) to alanines. Interestingly, none of these mutations affected the oligomerization of KicGAS (Supplementary Figure S4A). The changes also had little impact on DNA binding except for R97A which resulted in almost 10 fold reduction in binding affinity (Supplementary Figure S4B, Table 8). We have also performed the cGAS activity assay in presence of these mutants and found modest change in inhibition except for R97 (Table S3). The R97A mutation affected the inhibition to a large extent (IC_50_ = 6 μM versus WT IC_50_ of 0.9 μM) while the single mutation of other conserved residues (Supplementary Figure S2) had little to moderate effects (IC_50_ from 0.9 μM to 3 μM) in the inhibition of cGAS enzymatic activity.

### The basic residue cluster motif in the disordered C-terminal domain III of KicGAS is critical for DNA binding

**Figure 4.**
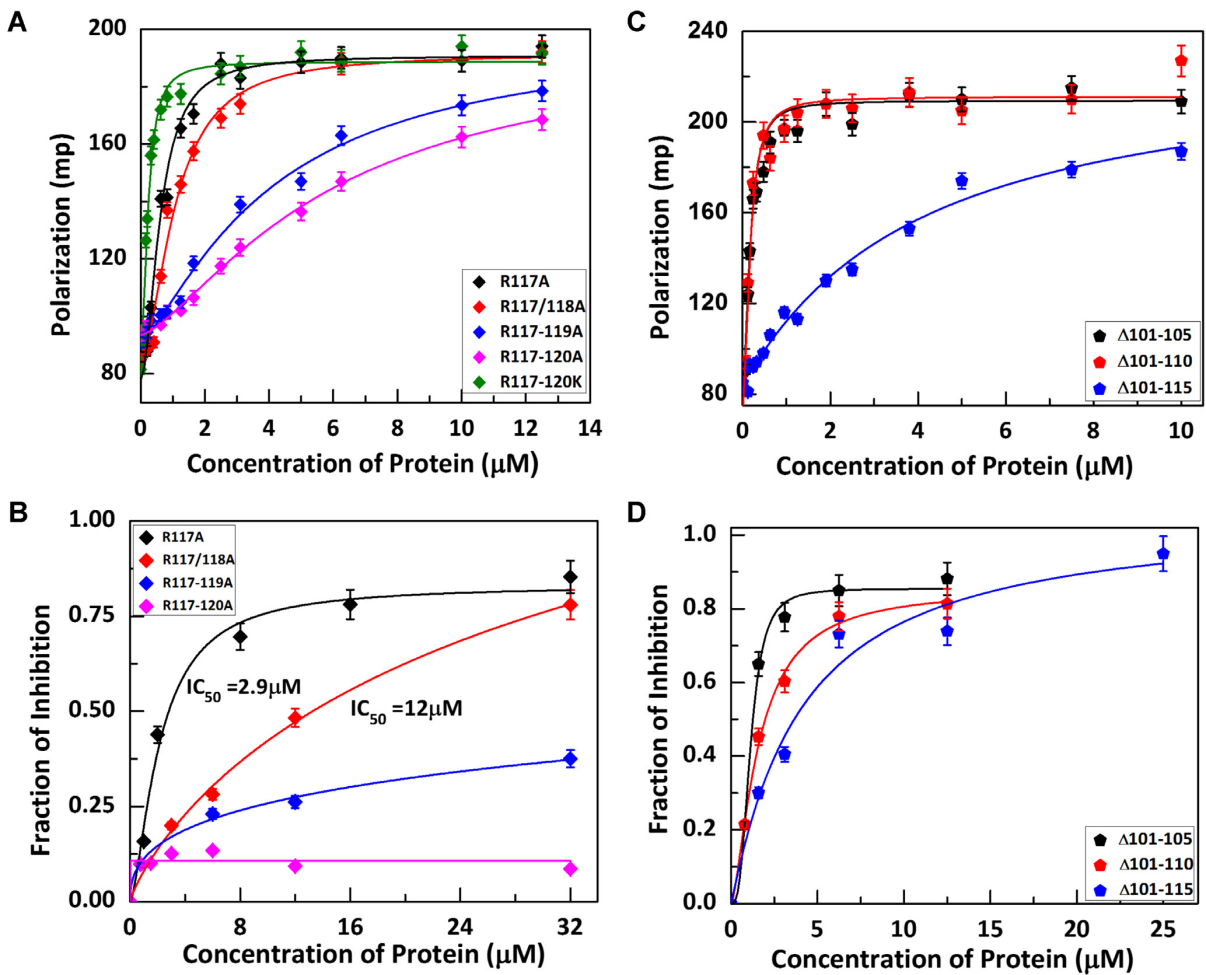
The positively charged cluster in the disordered domain III is crucial for DNA binding and cGAS inhibition. (**A**) Binding curves for the interaction of Arginine mutants in domain III with ISD45. (B) Fraction of inhibition of cGAS activity in presence different concentration of these mutants. (C, D) *The effect of the distance between the R-cluster (domain III) and the core domain on KicGAS DNA binding and cGAS inhibition*. (**C**) DNA binding curves for distance mutants obtained from Fluorescence Polarization experiment. (**D**) Fraction of inhibition of cGAS activity in presence different concentrations of distance mutants of KicGAS.

In contrast to domain I and II which are predicted to be structured, domain III appears to be disordered (Figure 3B). Although less conserved and varying in length, all corresponding regions in domain III of known γ herpesviruses contain a stretch of basic residue cluster (Supplementary Figure S2). As DNA is negatively charged, we speculated the positively charged cluster might be important for the DNA binding and thus cGAS inhibition. Indeed, the C-terminal truncation of the last 10aa had no effect on cGAS inhibition but truncation of additional 15 aa that included the basic residue cluster abolished the inhibition and DNA binding. To directly examine the roles of the R-cluster (^117^RRRR^120^), we mutated all these residues to alanines and observed that mutation of single, double, or triple arginine had little or partial effect, but changing all four together dramatically affected the DNA binding and cGAS inhibition (Figure 4A and B, Table 6), thus confirming the importance of the cluster in DNA binding. We also mutated all the four arginines to lysines and found no significant difference in DNA binding (Figure 4A), suggesting that the arginine residues are mostly involved in electrostatic interaction with DNA. Although domain III is apparently important for DNA binding, itself alone did not show substantial DNA binding even at the highest protein concentration we tested (100 μM in EMSA) (Supplementary Figure S5A). Consequently, domain III alone did not inhibit cGAS enzymatic activity (Supplementary Figure S5B). This suggests that domain III alone is not sufficient for DNA binding and that it must cooperate with domain I and II for optimal DNA binding and cGAS inhibition.

**Table 6.** Binding parameters for the interaction of ISD45 with domain III charge deficient mutants of FL KicGAS

	R117A	R117/118A	R117-119A	R117-120A	R117-120K
*K*_d_ (μM)	0.71 ± 0.04	1.01 ± 0.09	4.06 ± 0.70	5.88 ± 0.72	0.22 ± 0.01
n	1.97 ± 0.21	2.01 ± 0.37	1.31 ± 0.14	1.41 ± 0.10	1.79 ± 0.20

**Table 7.** Binding parameters for the interaction of ISD45 with domain I mutants of FL KicGAS

	L17A	I21A	L24A	E27A	N28A	L31A
*K*_d_ (μM)	0.25 ± 0.02	1.05 ± 0.30	3.39 ± 0.46	0.23 ± 0.02	1.12 ± 0.11	5.59 ± 1.0
n	1.48 ± 0.24	0.75 ± 0.08	1.15 ± 0.13	1.92 ± 0.26	1.29 ± 0.19	1.2 ± 0.49

**Table 8.** Binding parameters for the interaction of ISD45 with domain II mutants of FL KicGAS

	L55A	L62A	L62A/I70A/V74A	K68A/K69A	V74A	V87A/L91A	L96A/I98A	R97A
*K*_d_ (μM)	0.22 ± 0.02	0.29 ± 0.03	0.22 ± 0.20	0.57 ± 0.05	0.10 ± 0.01	0.15 ± 0.01	0.41 ± 0.11	1.94 ± 0.60
n	1.40 ± 0.22	1.40 ± 0.19	1.92 ± 0.25	2.52 ± 0.40	1.34 ± 0.22	2.13 ± 0.31	1.46 ± 0.46	1.03 ± 0.18

The linker between the conserved domain II and the positively charged residue cluster varies from 8aa to 25aa among the KicGAS homologues. We next examined whether the size of the linker influences the DNA binding and cGAS inhibition. For this purpose, we generated three mutants with increased deletion in the linker region (Δ101–105, Δ101–110, Δ101–115) and examined their binding with ISD45. We found that deletion of 5aa or 10aa had little impact on DNA binding (Figure 4C). However, if we delete the entire linker, the binding affinity to ISD45 decreased ∼26-fold (0.16 μM versus 3.89 μM) (Figure 4C) resulting in much reduced inhibition of cGAS (Figure 4D). During protein preparation, we also noticed a decrease in protein solubility. These results suggested the length of the linker is not critical as long as it reaches to minimum of ∼5aa. All together, these results suggested that the R-cluster in domain III is critical for its DNA binding and inhibition of cGAS and that a minimal flexible linker between the cluster and the core domain is required for optimal DNA binding and cGAS inhibition.

### KicGAS is capable of forming condensates with DNA in solution

**Figure 5.**
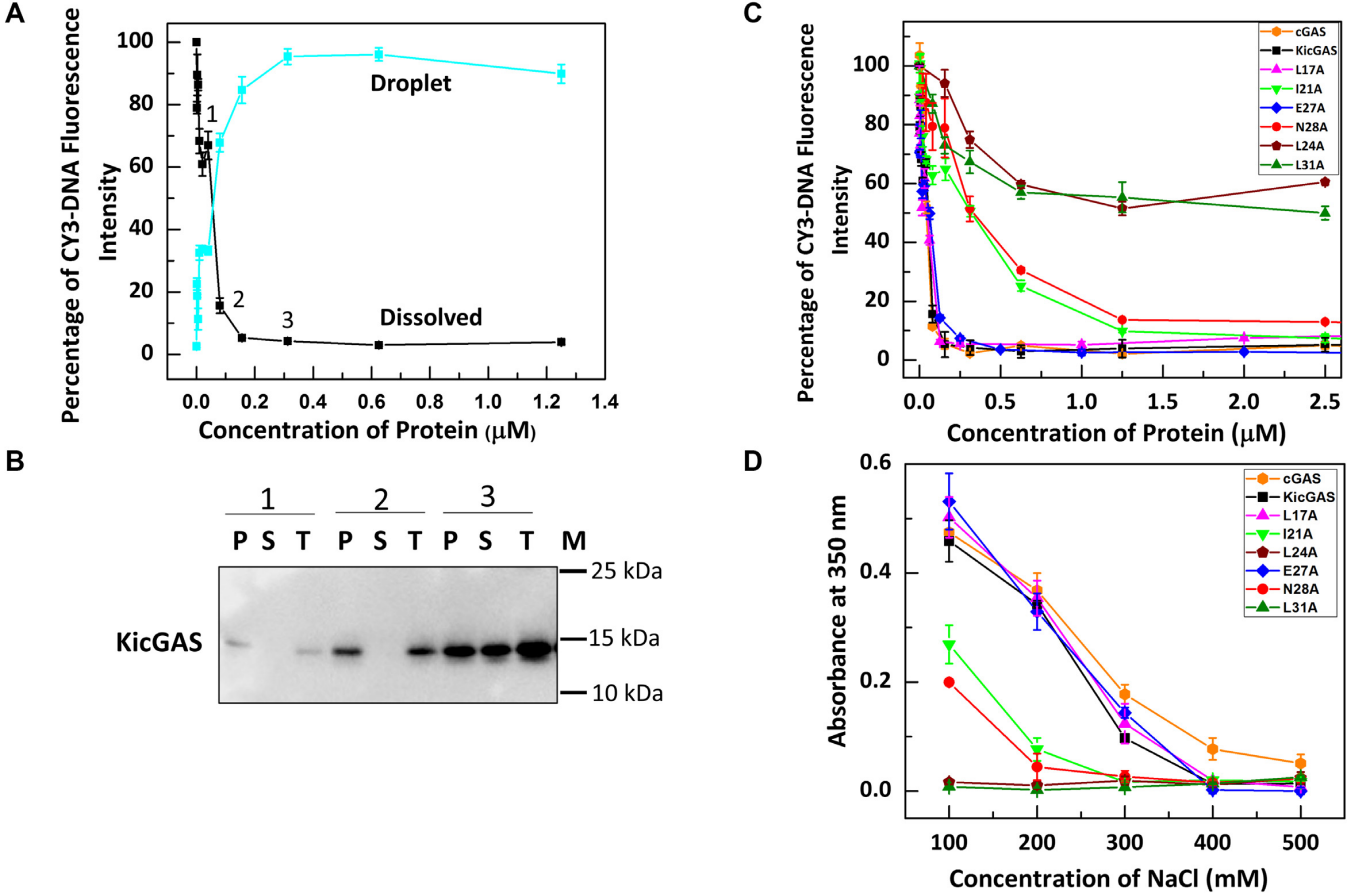
DNA binding promotes phase separation of KicGAS and DNA binding deficient mutants showed reduced ability of condensate formation. (**A**) DNA condensation assays of Cy3- ISD100 with KicGAS protein as monitored by spin down assay. (**B**) Western blot analysis for the supernatant and pellet obtained after spin-down assay. The number 1–3 corresponds to the respective samples in (A). T: Total protein before addition of DNA, S: Protein in the supernatant after spin down, P: Protein in the droplet after spin down, M: Protein size Markar (**C**) DNA condensation assay for all KicGAS domain I mutants with Cy3– ISD100. **(D)** The liquid-phase separation ability of KicGAS and its mutants as monitored by turbidity at different salt concentration.

Nucleic acids binding proteins can undergo phase separation with nucleic acids through multivalent interactions especially those involving intrinsically disordered region (IDR) (33). As the DNA binding of KicGAS depends on oligomerization as well as on presence of the intrinsically disordered domain III, which contributes to multivalent interactions of KicGAS binding to DNA, we speculated that KicGAS may phase separate upon DNA binding. Indeed, we observed that wild type full length KicGAS protein quickly turned milky when mixed with DNA (ISD100), a common phenomenon associated with protein phase separation such as cGAS upon binding to DNA (2,34). The formation of KicGAS-DNA condensates was monitored by measuring free DNA in the solution after separation of the condensates by centrifugation (34). The Cy3 fluorescence intensities remained in the supernatant after centrifugation decreased rapidly as the KicGAS concentration increased to 24 nM and dropped to the background level when KicGAS concentration reached 100 nM (Figure 5A). We have also confirmed that the lost fluorescence intensity in the supernatant could be recovered in the pellet upon redissolving in 1 M NaCl. Fluorescent intensity in the redissolved pellet increases as the KicGAS protein concentration increases and is inversely correlated with fluorescence intensity in the supernatant (Figure 5A). We also detected KicGAS protein by western blot and found that more protein was detected in the pelleted condensates than in the supernatant as KicGAS protein concentration increased (Figure 5B). We used cGAS as a control and found that WT KicGAS showed similar behavior to that of cGAS as well as to the curves of L17A and E27A. Remarkably, the decreases of the Cy3 fluorescence intensities in solutions of all oligomerization deficient mutants (I21A, L24A, N28A, L31A) were less dramatic than that of WT KicGAS under the same concentrations (20–100 nM) (Figure 5C). In addition, we also observed that the additive mutations of the R-residues to alanine in the disordered domain III significantly reduced the condensation (Supplementary Figure S6), implying that the oligomerization and the R-cluster are required for the DNA-induced phase separation of KicGAS. The condensation was also monitored by turbidity assays (34,35). The KicGAS–DNA turbidity measurements showed that turbidity was decreased when salt concentration increased, suggesting that KicGAS–DNA condensates were less formed when salt concentration increased, further implying that electrostatic interactions is the major driving force for the KicGAS–DNA phase separation (36) (Figure 5D). All the DNA binding deficient mutants showed reduced propensity to phase condensates formation as shown by reduced turbidity formation. Among those, the charge deficient mutant R117-120A and the oligomerization deficient mutant L24A and L31A showed most reduced turbidity, suggesting the role of these residues in promoting the formation of DNA induced condensates.

### KicGAS forms liquid droplets upon interacting with DNA

**Figure 6.**
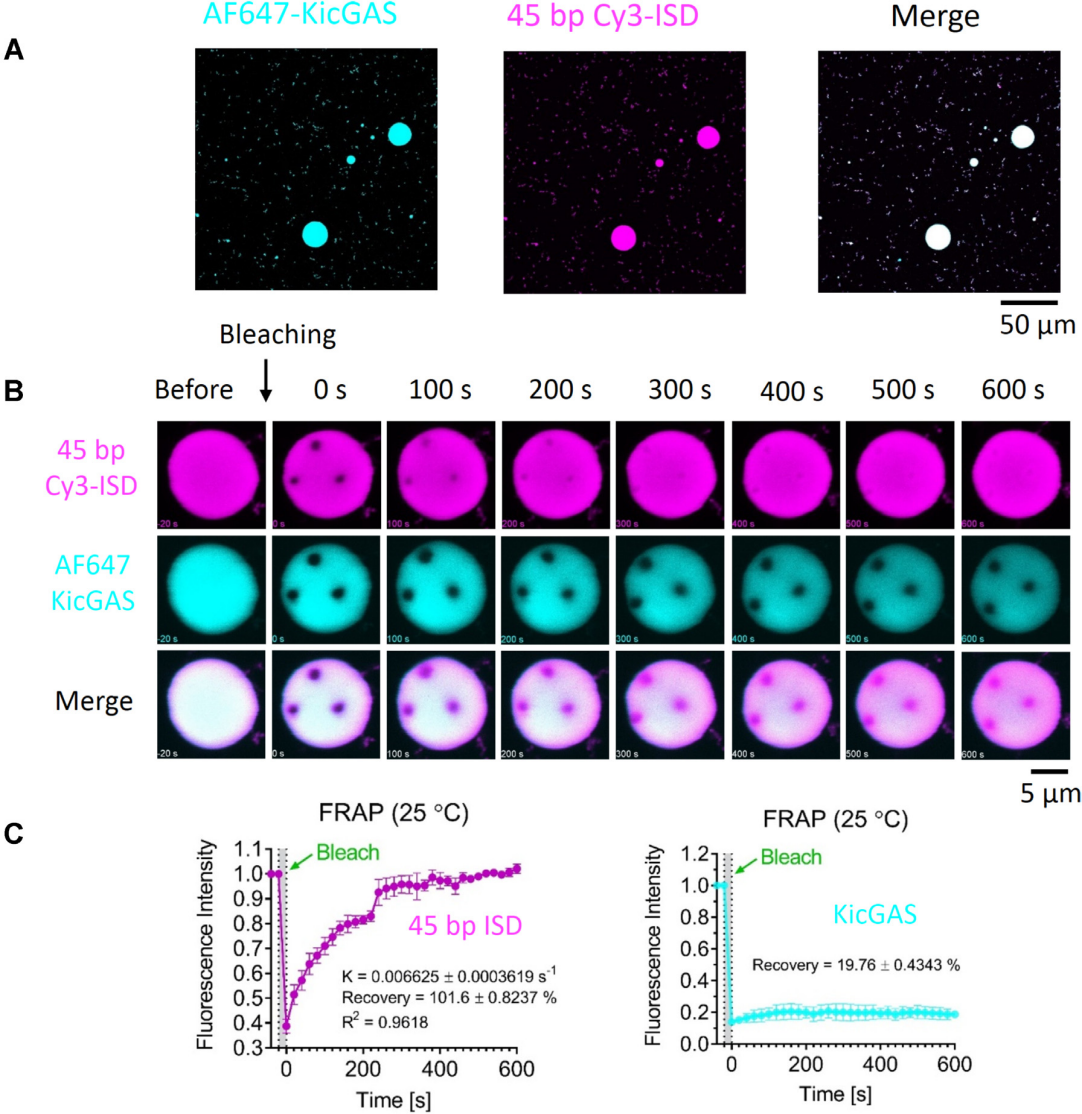
KicGAS underwent phase separation upon binding with DNA. (**A**) Representative fluorescent images of KicGAS–DNA phase separation. (**B**) Representative micrographs of FRAP experiments on partial bleaching of KicGAS–DNA condensates. (**C**) Quantification of FRAP of KicGAS–DNA liquid droplets in (B). Bleaching was performed at 30 min after KicGAS (30 μM) and DNA (30 μM) were mixed, and the recovery was allowed to occur at 25°C. Time 0 indicates the start of recovery after photobleaching. Shown are the means ± SD. *n* = 3 photobleaching bleaching areas in one liquid droplet.

To test whether DNA binding induces phase separation of KicGAS in vitro, we incubated fluorescently labeled KicGAS protein with Cy3 labeled ISD45. After mixing, WT KicGAS and ISD45 formed micron sized droplets, a typical feature of liquid phase separation (Figure 6A). Such DNA induced phase separation of KicGAS depends on DNA length. KicGAS underwent phase separation with DNA as short as 20 base pairs (bp) but not with 15 bp DNA (Supplementary Figure S7). This is consistent with the length-dependent DNA binding of KicGAS as observed (Fig 2C, Table 2). In addition, KicGAS mutants that showed lower affinity towards DNA, exhibited weakened phase separation (Supplementary Figure S8).

To assess the dynamics of KicGAS and DNA inside and outside the condensates, we performed a fluorescence recovery after photo bleaching (FRAP) assay. We found that the fluorescence of DNA was quickly recovered (K = 0.006625 ± 0.0003619 s^–1^) when partial photo bleaching was performed 30 min after the initiation of phase separation (Figure 6B and C, movie S1), which suggests that DNA exhibited liquid behavior inside the droplets. In contrast, the fluorescence recovery of KicGAS was much slower (Figure 6B and C, movie S1), which suggests that KicGAS exhibited solid-like behavior inside the droplets. The result suggests liquid nature of the droplets and interestingly higher mobility of DNA than KicGAS protein within the droplets. Full photo bleaching of either KicGAS or DNA showed low or no recovery of fluorescence, which suggests that KicGAS or DNA was less exchanged with molecules outside the droplets (Supplementary Figure S9, movie S2), and this perhaps is due to that KicGAS and DNA were sequestered into the droplets, leaving the solution free of KicGAS and DNA.

### KicGAS inhibits DNA induced phase separation of cGAS

**Figure 7.**
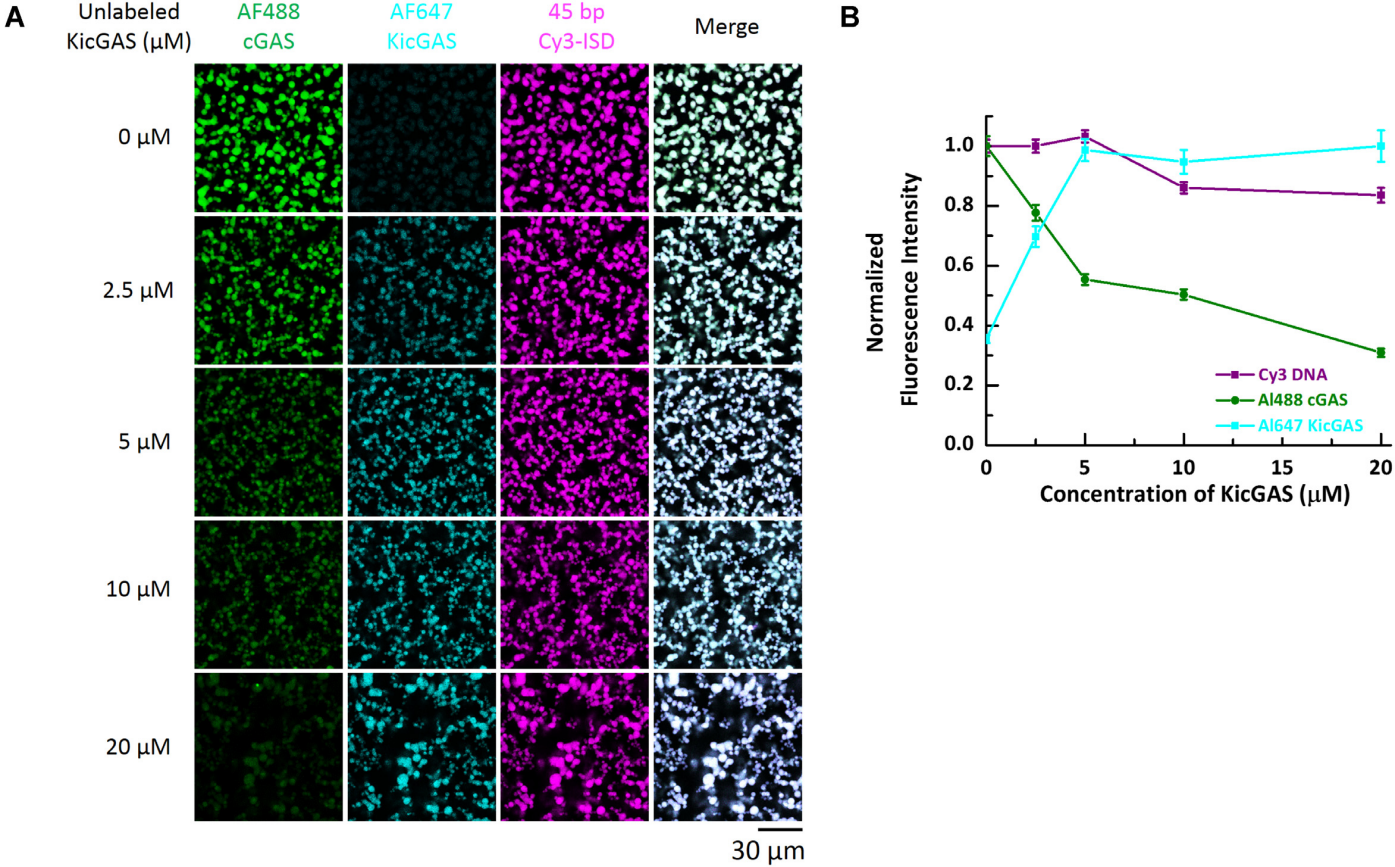
KicGAS competitively inhibited cGAS phase separation with DNA. (**A**) Representative fluorescent images of cGAS–DNA phase separation in the absence and presence of KicGAS at indicated concentrations. (**B**) Plot of Cy3 DNA, AF488 cGAS and AF647 KicGAS fluorescence intensities in the presence of increasing concentrations of KicGAS. Data were normalized by maximum value to 1. Values shown are means ± standard error. In both (A) and (B), cGAS: 10 μM, 3% Alexa Fluor 488-labeled; DNA: 10 μM, 2% Cy3-labeled. 200 nM Alexa Fluor 647 labeled KicGAS was supplemented for each titration.

**Figure 8.**
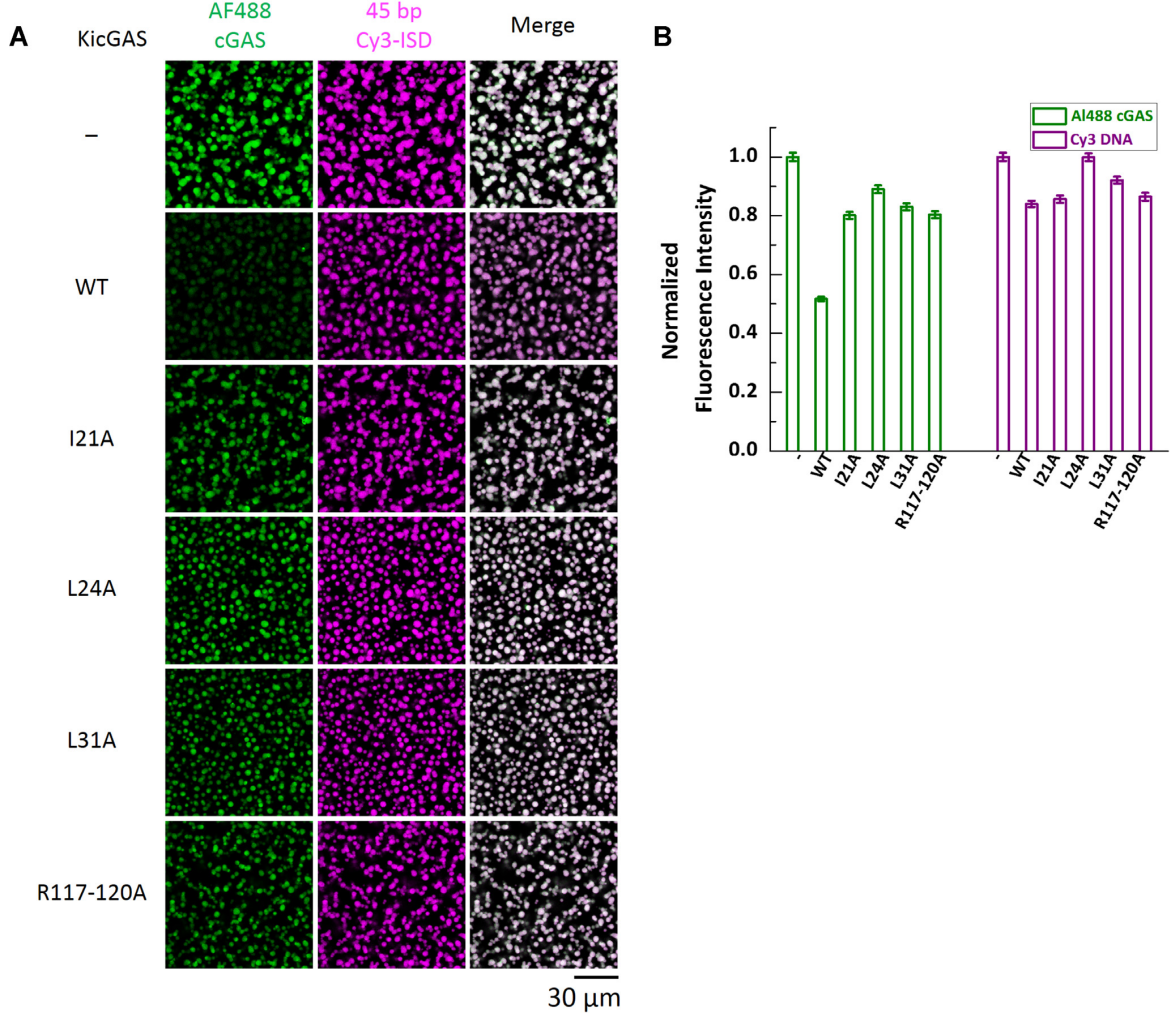
DNA binding deficient mutants of KicGAS have less inhibition on cGAS phase separation with DNA than WT KicGAS. (**A**) Representative fluorescent images of cGAS–DNA phase separation in presence of different mutants of KicGAS (10 μM). cGAS: 10 μM, 3% Alexa Fluor 488-labeled; 45-bp ISD: 10 μM, 2% Cy3-labeled. AF488, Alexa Fluor 488. (**B**) Quantification plot of AF488 cGAS and Cy3 DNA fluorescent intensities in (A). Data were normalized by maximum value to 1. Values shown are means ± standard error.

DNA binding to cGAS robustly induces the formation of liquid-like droplets in which cGAS is activated and the conditions that affect cGAS phase transition also impact its activity (2,34). Because KicGAS also underwent phase separation with DNA, we next examined whether KicGAS influences DNA-induced phase separation of cGAS. We mixed constant concentrations of ISD45 (10 μM, 2% was labeled with Cy3) and cGAS (10 μM, 3% was labeled with Alexa Fluor 488) with none or increasing concentrations of KicGAS and monitored the fluorescence signals using a confocal fluorescent microscope. To visualize KicGAS, we included 200 nM Alexa Fluor 647 labeled KicGAS (AF647-KicGAS) with unlabeled KicGAS in each titration concentration. Overall, we found that as the concentration of KicGAS increased, the fluorescent intensities of cGAS decreased and less cGAS was enriched in the droplets while the fluorescent intensities of ISD45 showed little change (Figure 7A and B). These results suggested that KicGAS competitively inhibits DNA-induced cGAS phase separation. We repeated the assays with the KicGAS mutants that have reduced propensity to condensates formation. We found less changes in the fluorescence intensity of AF488-cGAS inside the liquid droplet in the presence of L24A, I21A, L31A or R117-120A (Figure 8). These results further support that KicGAS competitively inhibits DNA-induced cGAS phase separation and highlight the importance of oligomerization and DNA binding of KicGAS in inhibition of DNA induced phase separation of cGAS.

## DISCUSSION

Since the discovery of cGAS, an increasing number of viral factors have been described to interfere with the cGAS–cGAMP–STING pathway, highlighting the critical roles of this pathway in the host antiviral defenses (23,37–42). Among these viral factors, most target the downstream components of the pathway, KicGAS is the first reported inhibitor to directly inhibit cGAS activity. The aim of this study was to elucidate the mechanisms by which KicGAS inhibits cGAS. Our extensive biochemical and functional analyses of KicGAS have revealed the DNA binding characteristics of KicGAS, and identified its critical residues, and structural features that required for cGAS inhibition. We found that KicGAS protein forms oligomer and binds to DNA cooperatively and that optimal DNA binding of KicGAS requires both structured and disordered regions, and their coordination. We also found that KicGAS likewise forms liquid droplets upon binding to DNA and inhibits DNA-induced phase separation of cGAS, a critical step for cGAS activation.

Our previous studies revealed that DNA binding of KicGAS is important for cGAS inhibition, we therefore first characterized the DNA binding properties in more detail. We revealed that KicGAS binds to DNA in a sequence independent but length dependent manner, which is similar to cGAS. We also confirmed our earlier observation that KicGAS binding to DNA is no better than cGAS binding to ISD45 (*K*_D_ = 150 versus 50 nM). Because no known DNA binding domain has been identified in KicGAS, we sought to map domains required for DNA binding and cGAS inhibition. We found near full length protein (aa9–121) of KicGAS is required for cGAS inhibition, suggesting that structural integrity of KicGAS is needed for its function. Interestingly, we found that KicGAS binds to DNA cooperatively with Hill coefficient of 2.1 (for ISD45) and the Hill coefficient increases with increase in DNA length similar to cGAS (28). The cooperativity is conceivably mediated by oligomerization of KicGAS because oligomerization deficient mutants resulted in much reduced DNA binding and cGAS inhibition.

Despite binding to DNA, no known DNA binding domain could be identified based on KicGAS amino acids sequence, and KicGAS shares no homology to any cellular proteins. Multiple alignment of KicGAS homologues of γ herpesviruses revealed three putative domains, N-terminal domain I, middle region domain II, and C-terminal domain III. While domain I and domain II share significant sequence homology among the homologues of γ herpesviruses, domain III is less conserved and predicted to be disordered. We found that deletion of any of the three domains compromised KicGAS DNA binding and cGAS inhibition, suggesting all three domains are required for DNA binding and cGAS inhibition.

Because all examined KicGAS homologues of γ herpesviruses inhibited cGAS, we first examined the conserved residues in Domain I and II. Among five invariably conserved residues in all KicGAS homologues, four of them (L24, E27, N28, L31) are located within domain I. We mutated each of them and a few other highly conserved hydrophobic residues (L17, I21) to alanine. We found that mutation of I21, L24, N28 or L31 disrupted oligomerization of KicGAS, consequently loss of DNA binding and cGAS inhibition. These results suggest that the conserved hydrophobic residues in domain I are involved in oligomerization of KicGAS and that KicGAS oligomerization is critical for DNA binding and cGAS inhibition. Domain II is not only conserved among γ herpesviruses but also structurally related although distantly to a region in VP22 of α herpesviruses (32). We similarly mutated all the conserved residues in domain II of KicGAS individually. However, none of these mutations altered the size exclusion chromatography profiles of the purified proteins, suggesting no effect of these mutations on protein oligomerization. These mutations also had little effects on KicGAS DNA binding or cGAS inhibition. These results suggested the intermolecular interaction mediated by domain II is rather strong and quite stable. In contrast to structured domain I and II, domain III appears to be intrinsically disordered (Figure 3B). A distinct feature of domain III among both γ1 and γ2 homologues is a cluster of positively charged residues. Deletion of the R-cluster abolished KicGAS binding to DNA suggesting that the positively charged residues in this domain contribute critically to DNA binding. Although required, domain III alone was unable to bind with DNA, suggesting a coordination with structured domains I and II is required for efficient DNA binding and cGAS inhibition.

Although the structure of KicGAS was not available, the crystal structure of its homologue of MHV68 has been solved (43). The overall structure consists of 3 α helices and 1 β sheet. Domain I consists of one α helix (α1), domain II consists of a long and short α helix (α2 and α3 respectively), and a short beta sheet (β). The first α-helix in domain II (α2) interacts with α2 of other monomer with reverse orientation to form a dimer and expose the first α1 in domain I at either ends. The dimeric form of the protein is entirely contributed by α2 and independent of α1 helix. Moreover, the dimers preferentially form a tetramer mediated by interactions between the α1 of each dimer. Our observations fit well with these structural features: (i) the oligomerization is mostly mediated by domain I; (ii) the domain II is mostly involved in strong and stable dimeric interactions; (iii) domain III was excluded from the structure presumably because of its intrinsic disorder. By analogy to the structure of MHV68 homologue, we speculate KicGAS dimerizes through stable intermolecular interaction mediated by α2 in domain II and this dimer further oligomerizes through the interaction between the α1 in domain I. It is noteworthy to mention that disordered domain III is not involved in oligomerization but contribute greatly to DNA binding via the cluster of positively charged residues. Both oligomerization mediated by the structured region and the cluster of positively charge residues are required for KicGAS DNA binding and cGAS inhibition.

cGAS undergoes robust DNA induced phase transition resulting the formation of liquid like droplets, in which the reactants are concentrated and cGAS catalyzes the formation of cGAMP. Multivalent interaction between the positively charged DNA binding surface of cGAS and the negatively charged DNA triggers cGAS a switch-like response when the DNA concentration reaches a certain threshold. Such a switch-like response is enhanced in presence of longer DNA leading to efficient production of cGAMP (2). In addition to the impact on cGAS enzymatic activity, cGAS-DNA phase separation has also been recently shown to protect DNA from nuclease TREX1 degradation in cells (26). Similar to cGAS, KicGAS also recognizes DNA in a length dependent manner and DNA binding leads to the efficient formation of phase separated liquid droplets. We found that KicGAS phase separation upon DNA binding requires oligomerization of KicGAS and the C-terminal intrinsically disordered region especially the positively charged R-cluster that presumably mediate collective multivalent interactions with DNA. In addition, KicGAS competes with cGAS for DNA binding and suppresses DNA-induced phase separation of cGAS and thereby inhibiting cGAS-DNA sensing. Our studies revealed a great example how viral factors directly inhibit the DNA sensor cGAS.

The direct viral inhibitors of cGAS remained scarce (23,42). It is unclear how prevalent a similar mechanism is used by other viruses. Because KicGAS is conserved among γ herpesviruses and distantly related to VP22 of α herpesviruses, we speculate this mechanism could be used by other herpesviruses. Indeed, while our manuscript was in revision, Xu *et al.* reported that KicGAS and its homologues of both γ and α herpesviruses efficiently restrict cGAS-DNA phase separation both in vitro and in cells (44). Although viral inhibitors of cGAS remain relatively rare, there are numerous direct inhibitors of RNA sensors with enzymatic activities such as RIG-I (helicase, ATPase), PKR (kinase) and OAS (homologue to cGAS). Most of the viral inhibitors of RNA sensors are oligomeric RNA-binding proteins, reminiscent of structural features of KicGAS. The examples include non-structural protein 1 (NS1) from Influenza A virus (IAV), viral protein 35 (VP35) from Ebola virus (EBOV), and Marburg virus, as well as the E3L protein from Vaccinia virus (45–47). Structural studies have revealed that the RNA binding domain (RBD) and the effector domain (ED) of one NS1 dimerizes with adjacent NS1 resulting a chain of polymer-like structures that form a tubular structure with a tunnel to fit dsRNA, thus hiding dsRNA from RIG-I (48). VP35 contains N-terminal oligomerization domain and a C-terminal dsRNA binding domain and deletion of either the oligomerization domain or RNA-binding domain (RBD) diminishes VP35 mediated inhibition of RIG-I RNA-sensing and downstream signaling (49). These mechanisms appear similar to those used by KicGAS because inhibition of their targeted nucleic acid sensors requires both binding to nucleic acids PAMPs and self oligomerization. Structural work has demonstrated that RNA sensors RIG-I and MDA5 polymerize upon binding RNAs (50–53). Interestingly, these RNA sensors were shown to colocalize with stress granules (54) while sensing RNA and stress granules are another example of phase separation (55). It will be fascinating to know whether RNA sensors and their RNA-binding viral inhibitors underwent phase separation upon engaging with RNAs to ensure more efficient sensing or inhibition. Although more detailed mechanisms by which KicGAS inhibits cGAS awaits further structural characterizations, these observations suggest viruses may have evolved similar mechanisms to target the nucleic acids sensors and prevent them from being activated.

## Supplementary Material

gkab689_Supplemental_Files

## Notes

Present address: Jianjun Wu, Lerner Research Institute, 9500 Euclid Avenue, Cleveland, Ohio 44195, USA.
