# Supplementary material for: Cooperative DNA binding mediated by KicGAS/ORF52 oligomerization allows inhibition of DNA-induced phase separation and activation of cGAS
Source: Nucleic Acids Res. 2021 Aug 13;49(16):9389–403. doi: 10.1093/nar/gkab689 (PMC8450086; doi:10.1093/nar/gkab689)
Supplement: gkab689_Supplemental_Files [file gkab689_supplemental_files.zip › suplimentary Information.pdf]

Table S1: Nucleic acids used in this study*	
ISD12	5’-ACTGATCTGTAC-3’<br>3’-TGACTAGACATG-5’
ISD20	5’-ATCTATGACTGATCTGTACA-3’<br>3’-TAGATACTGACTAGACATGT-5’
ISD25	5’-CTAGTGATCTATGACTGATCTGTAC-3’<br>3’-GATCACTAGATACTGACTAGACATG-5’
ISD30	5’-ATCTACTAGTGATCTATGACTGATCTGTAC-3’<br>3’-TAGATGATCACTAGATACTGACTAGACATG-5’
ISD45	5’-TACAGATCTACTAGTGATCTATGACTGATCTGTACATGATCTACA-3’<br>3’-ATGTCTAGATGATCACTAGATACTGACTAGACATGTACTAGATGT-5’
Hsv60	5’-TAAGACACGATGCGATAAAATCTGTTTGTAAAATTTATTAAGGGTACAAATT-3’<br>3’-ATTCTGTGCTACGCTATTTTAGACAAACATTTTAAATAATTCCCATGTTTAA-5’
VACV72	5’-ATCCATCAGAAAGAGGTTTAATATTTTTGTGAGACCATCGAAGAGAGAAAGAGATAAAACTTTTTTACGACT-3’<br>3’-TAGGTAGTCTTTCTCCAAATTATAAAAACACTCTGGTAGCTTCTCTCTTTCTCTATTTTGAAAAAATGCTGA-5’
KS90	5’-TCGTCTCGCCTGTCAAATCGTCTATGTTTTTCGGCGCGTTGTGCCAATATAACTCTAGAAACTAATACTGTATCTGTCAGCAGTACTACA-3’<br>3’-AGCAGAGCGGACAGTTTAGCAGATACAAAAAGCCGCGCAACACGGTTATATTGAGATCTTTGATTATGACATAGACAGTCGTCATGATGT-5’
ssISD12	5’-ACTGATCTGTAC-3’
ssISD20	5’-ATCTATGACTGATCTGTACA-3’
ssISD25	5’-CTAGTGATCTATGACTGATCTGTAC-3’
ssISD30	5’-ATCTACTAGTGATCTATGACTGATCTGTAC-3’
ssISD35	5’-TACAGATCTACTAGTGATCTATGACTGATCTGTAC-3’
ssISD45	5’-TACAGATCTACTAGTGATCTATGACTGATCTGTACATGATCTACA-3’
ssHsv60	5’-TAAGACACGATGCGATAAAATCTGTTTGTAAAATTTATTAAGGGTACAAATT-3’
ssVACV72	5’-ATCCATCAGAAAGAGGTTTAATATTTTTGTGAGACCATCGAAGAGAGAAAGAGATAAAACTTTTTTACGACT-3’

90% GC	5’-CCGCCAGCCCGCGGGCTGGC-3’<br>3’-GGCGGTCGGGCGCCCGACCG-5’
0% GC	5’-ATTTAATAATAATTTATTAT-3’<br>3’-TAAATTATTATTAAATAATA-5’
30%GC	5’-ATATCGCTATAGATATCATC-3’<br>3’-TATAGCGATATCTATAGTAG-5’
45%GC	5’-CCATCAAAGAGAGAAAGAGC-3’<br>3’-CCATCAAAGAGAGAAAGAGC-5’
ssKS90	5’-<br>TCGTCTCGCCTGTCAAATCGTCTATGTTTTTCGGCGCGTTGTGCCAATATAACTCTAGAAACTAATACTGTATCTG<br>TCAGCAGTACTACA-3’
dsRNA45	5’-UACAGAUCUACUAGUGAUCUAUGACUGAUCUGUACAUGAUCUACA-3’<br>3’-AUGUCUAGAUGAUCACUAGAUACUGACUAGACAUGUACUAGAUGU-5’
DNA-RNA hybrid	5’-UACAGAUCUACUAGUGAUCUAUGACUGAUCUGUACAUGAUCUACA-3’<br>3’-ATGTCTAGATGATCACTAGATACTGACTAGACATGTACTAGATGT-5’
ssRNA45	5’-UACAGAUCUACUAGUGAUCUAUGACUGAUCUGUACAUGAUCUACA-3’
Cy3DNA 45	5’Cy3-TACAGATCTACTAGTGATCTATGACTGATCTGTACATGATCTACA-3’<br>3’-ATGTCTAGATGATCACTAGATACTGACTAGACATGTACTAGATGT-5’
*Nucleic Acids used in the polarization study and EMSA are FAM labelled at the 5’ end of the sense strand.	

Table S2: Strictly (blue) and highly conserved residues (black) of each domain																
	Domain I (D1)						Domain II (D2)									
	α-1						α-2			α-3		β				
KicGAS	17 L	21 I	24 L	27E	28N	31L	55L	62L	70I	74V	87V	91L	96L	97R	98I	100V
Alignment Score (all homologue ORF52)	9	8	10	10	10	10	8	8	8	8	5	8	6	10	8	7

Table S3: IC$_{50}$ Values of KicGAS mutants for the inhibition of cGAS activity and the corresponding K_d values for the interaction with ISD45.															
Mutants	L17A	I21A	L24A	E27A	N28A	L31 A	L55A	L62A	L62A/ I70A/ V74A	V74A	K68A /K69 A	V87A /L91A	L96A/I98A	R97A	R117-120A
IC$_{50}$	1.0±0. 17	6.47± 0.856	≥25	1.3±0.3 4	6.33± 0.93	≥25	0.92± 0.12	1.24±0. 09	2.29± 0.39	1.83± 0.22	2.64± 0.49	2.08± 0.22	3.17±0.80	5.5±0.5	≥25
K_d (μM)	0.25± 0.02	1.05± 0.30	3.39± 0.46	0.23±0. 02	1.12± 0.11	5.59 ±1.0	0.22± 0.02	0.29±0. 03	0.22± 0.20	0.10± 0.01	0.57± 0.05	0.15± 0.01	0.41±0.11	1.94±0.60	5.88±0.72

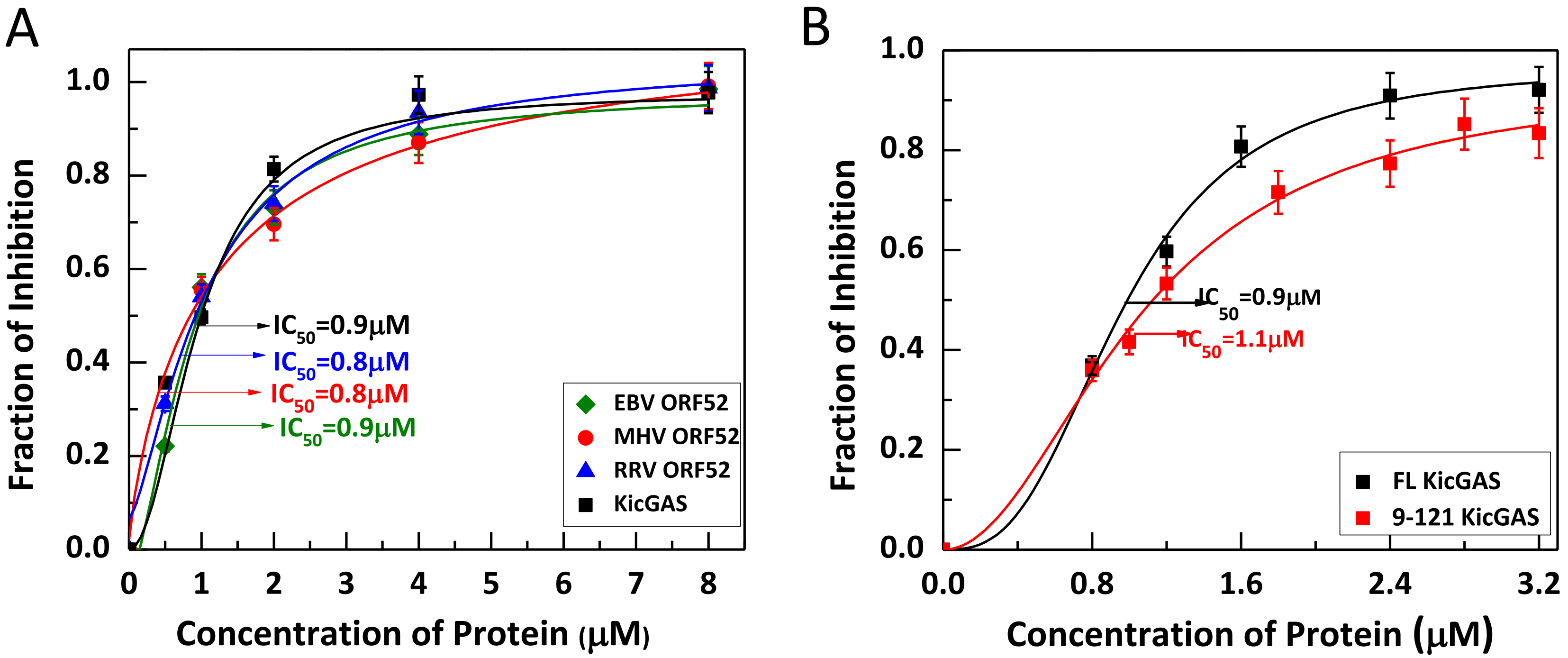

Figure S1. Inhibition of cGAS is evolutionarily conserved among γ-herpesvirus and nearly FL KicGAS (9-121) is required for optimum inhibition. Plot of the fraction of inhibition of cGAS activity in presence of different concentration of (**A**) KicGAS homologues, (**B**) FL KicGAS and 9-121 KicGAS.

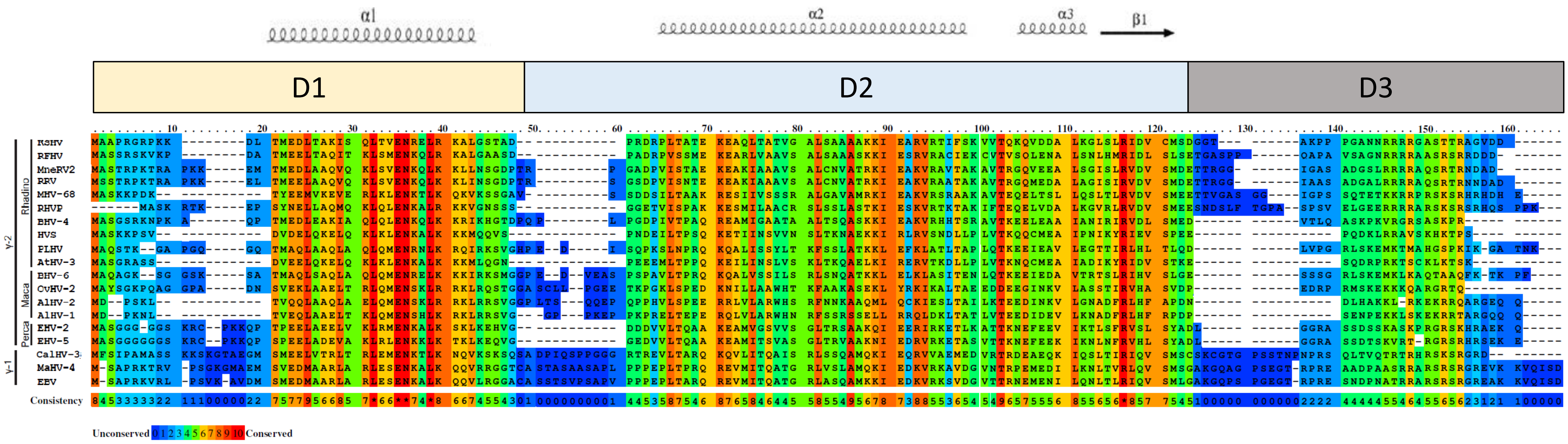

Figure S2. Multiple sequence alignment of gamma herpes virus ORF52 proteins. The three domains D1, D2 and D3 have been designed based on the secondary structure element of MHV68 ORF52.

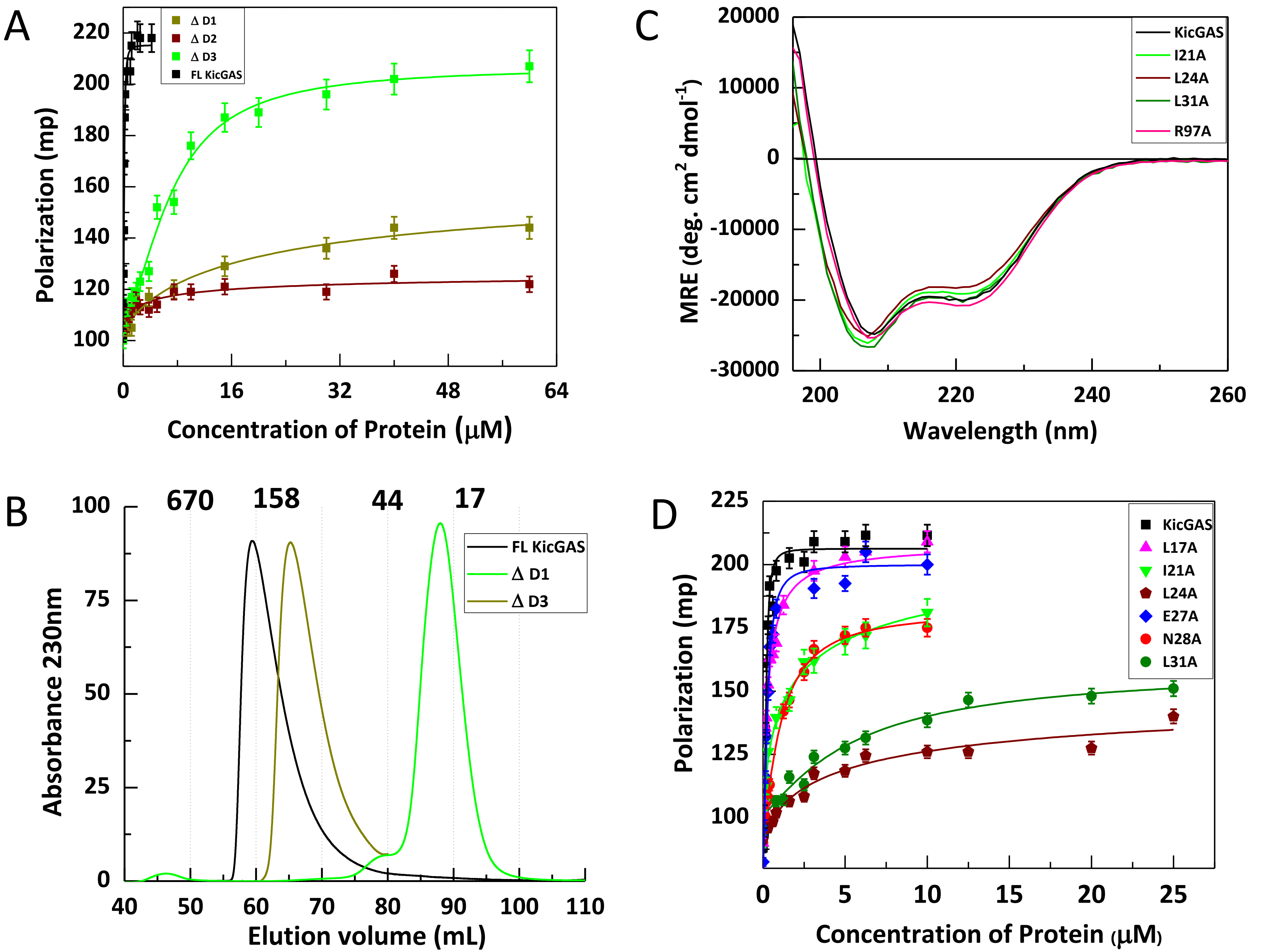

Figure S3. All the three domains are required for efficient DNA binding and Domain I is critical for oligomerization. Oligomerization deficient mutant and R97A does not cause misfolding but disrupts the DNA binding (**A**) DNA binding curves for ΔD1, ΔD2 and ΔD3 in comparison to WT KicGAS. (**B**) Comparison of the size exclusion chromatograph of the FL KicGAS, ΔD1 and ΔD3 protein. (Hiload 16/600 Superdex 200pg) (**C**) Circular dichroic spectra of KicGAS, I21A, L24A, L31A and R97A.(**D**) DNA binding curves for domain I mutants

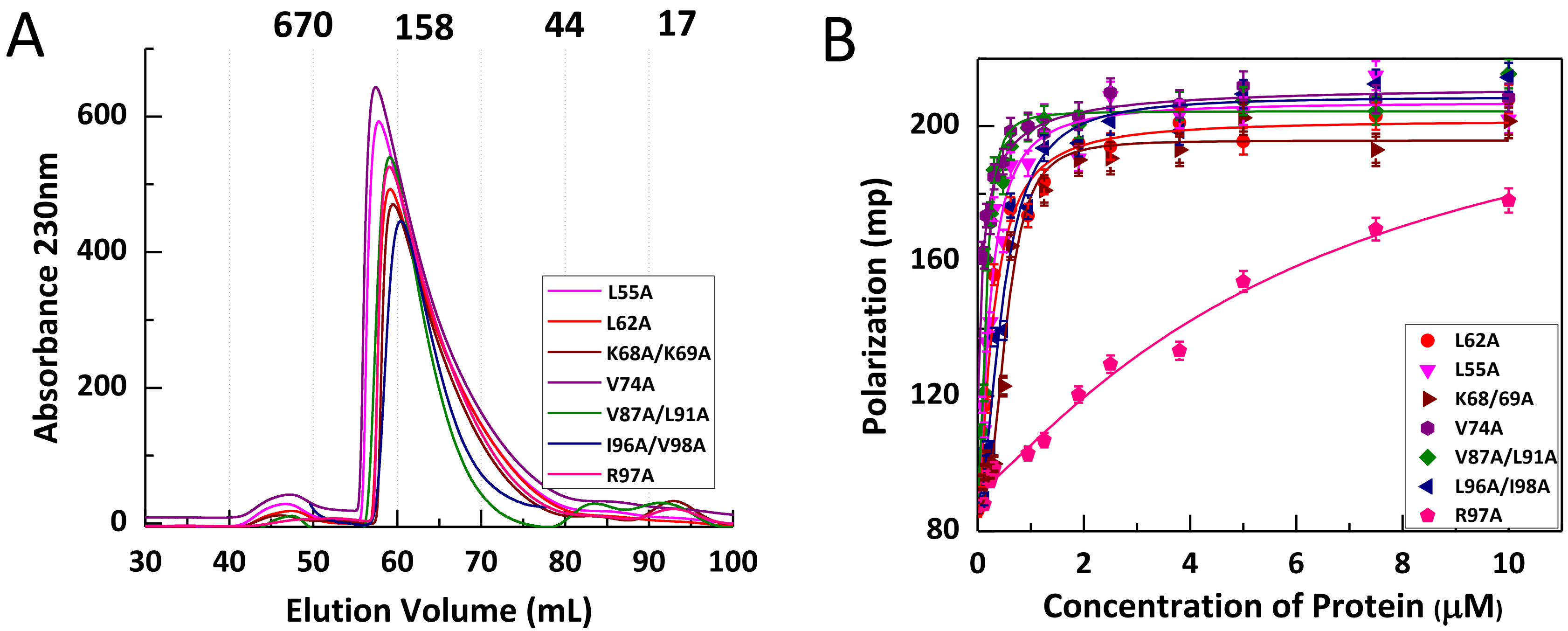

Figure S4. R97A in Domain II is important for DNA binding. Domain II mutants do not cause any change in oligomerization. (**A**) Size exclusion chromatographs of the domain II mutants(Hiload 16/600 Superdex 200pg) (**B**) Binding curves for the interaction of ISD45 with domain II mutants obtained from fluorescence polarization experiments.

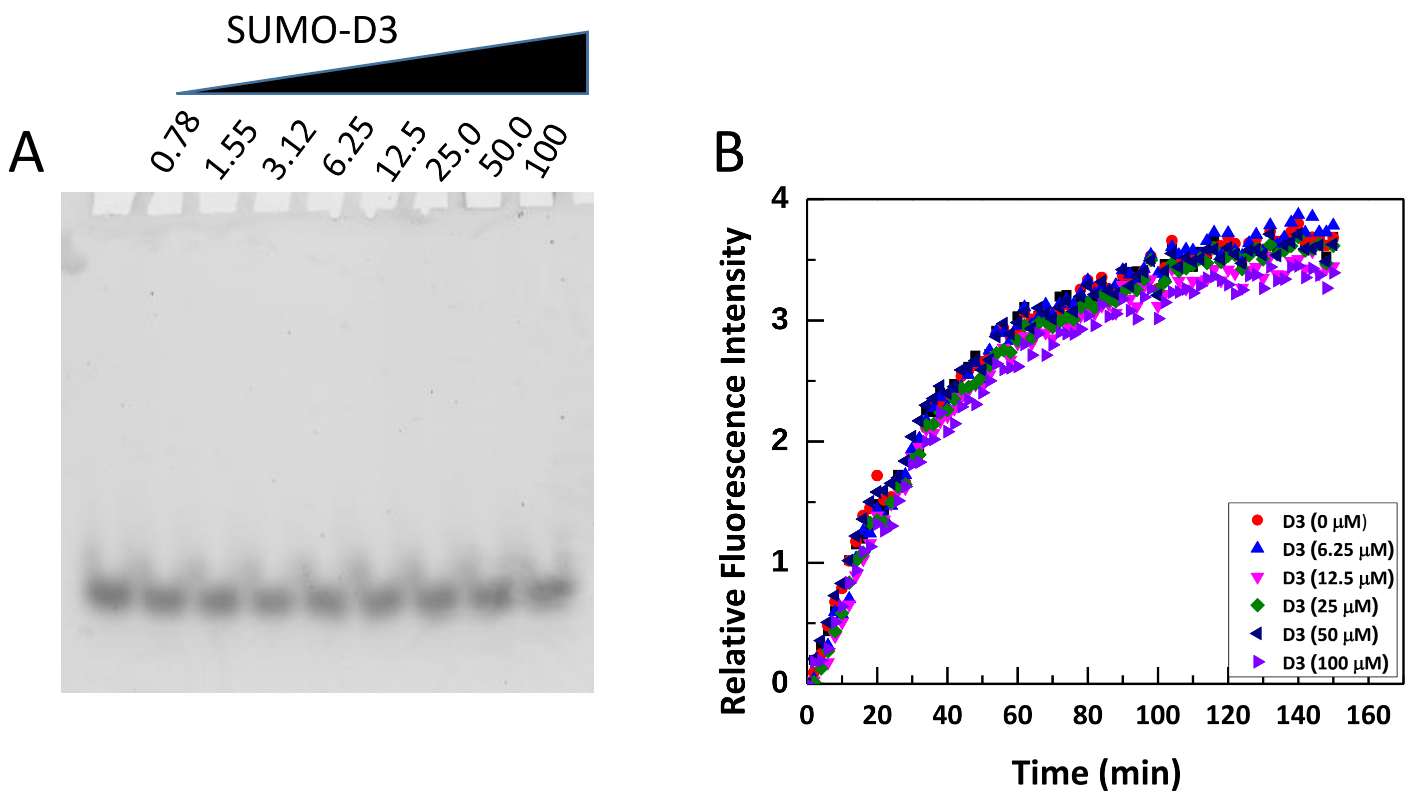

Figure S5. The disordered domain D3 itself cannot bind to DNA or inhibit cGAS. Result of (**A**) EMSA for the interaction of SUMO-D3 with ISD45 and (**B**) cGAS activity assay performed in presence of different concentration of D3.

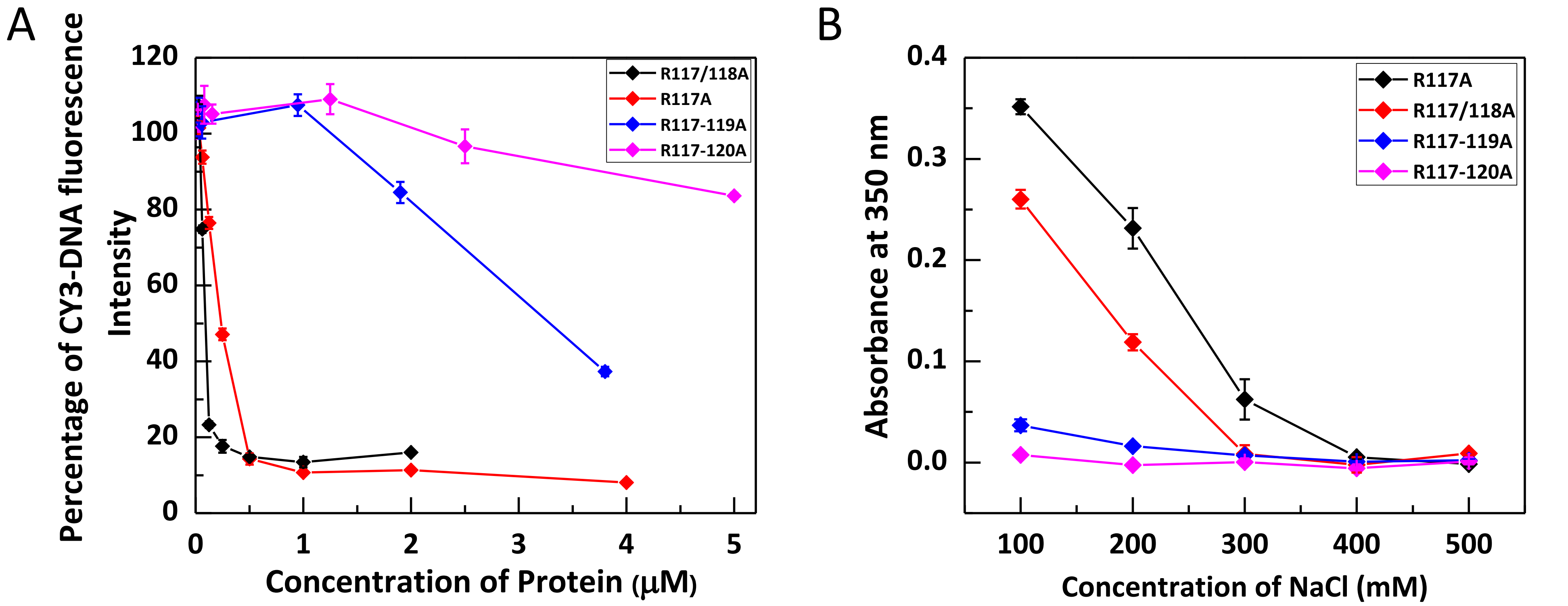

Figure S6. DNA binding promotes liquid phase condensation as monitored by spin down and turbidity assay. DNA condensation assays of Cy3-ISD100 with Arginine mutants in domain III as monitored by (**A**) spin down assay and (**B**) turbidity assay.

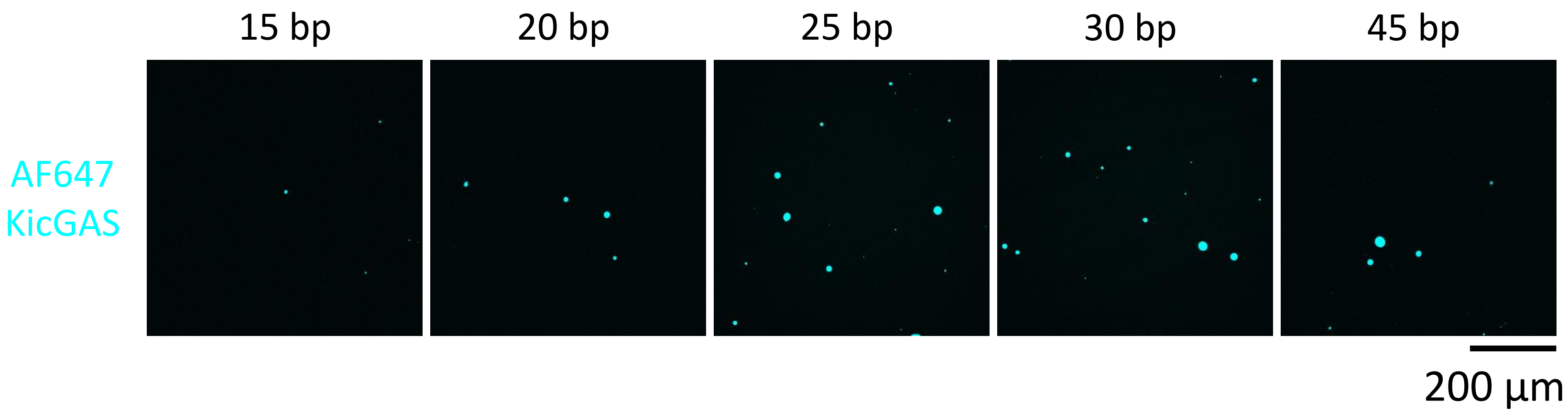

Figure S7. KicGAS can undergo phase separation with DNA having length 20bp or higher. Fluorescence images of phase separation by mixing of DNA of different lengths as indicated with full-length KicGAS. The images shown are representative of all fields in the wells.

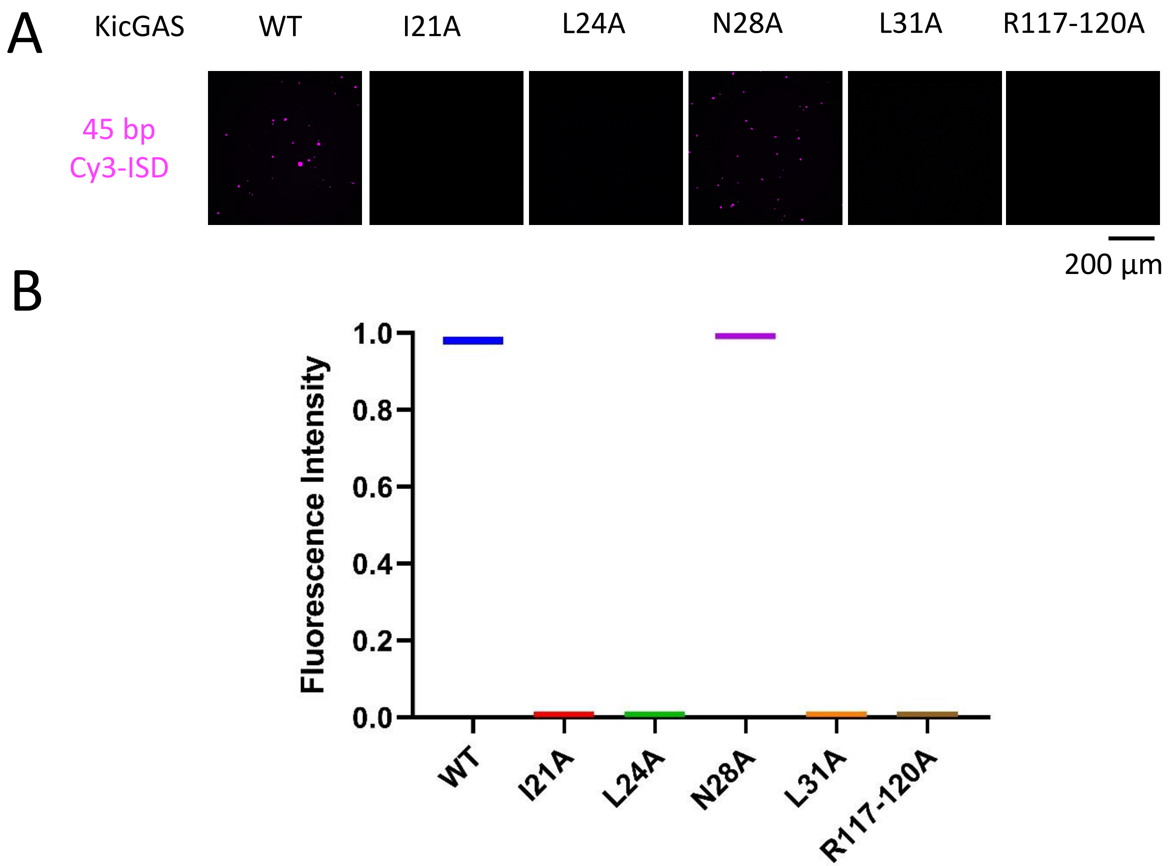

Figure S8. KicGAS mutations with low affinity to DNA exhibits weakened phase separation with DNA. (**A**) Representative fluorescence images of DNA induced phase separation of KicGAS and its mutants. (**B**) Plot of fluorescence intensities for DNA induced phase separation of KicGAS and its mutants in (**A**). Data were normalized to 1 by wild type KicGAS. n = 1 image.

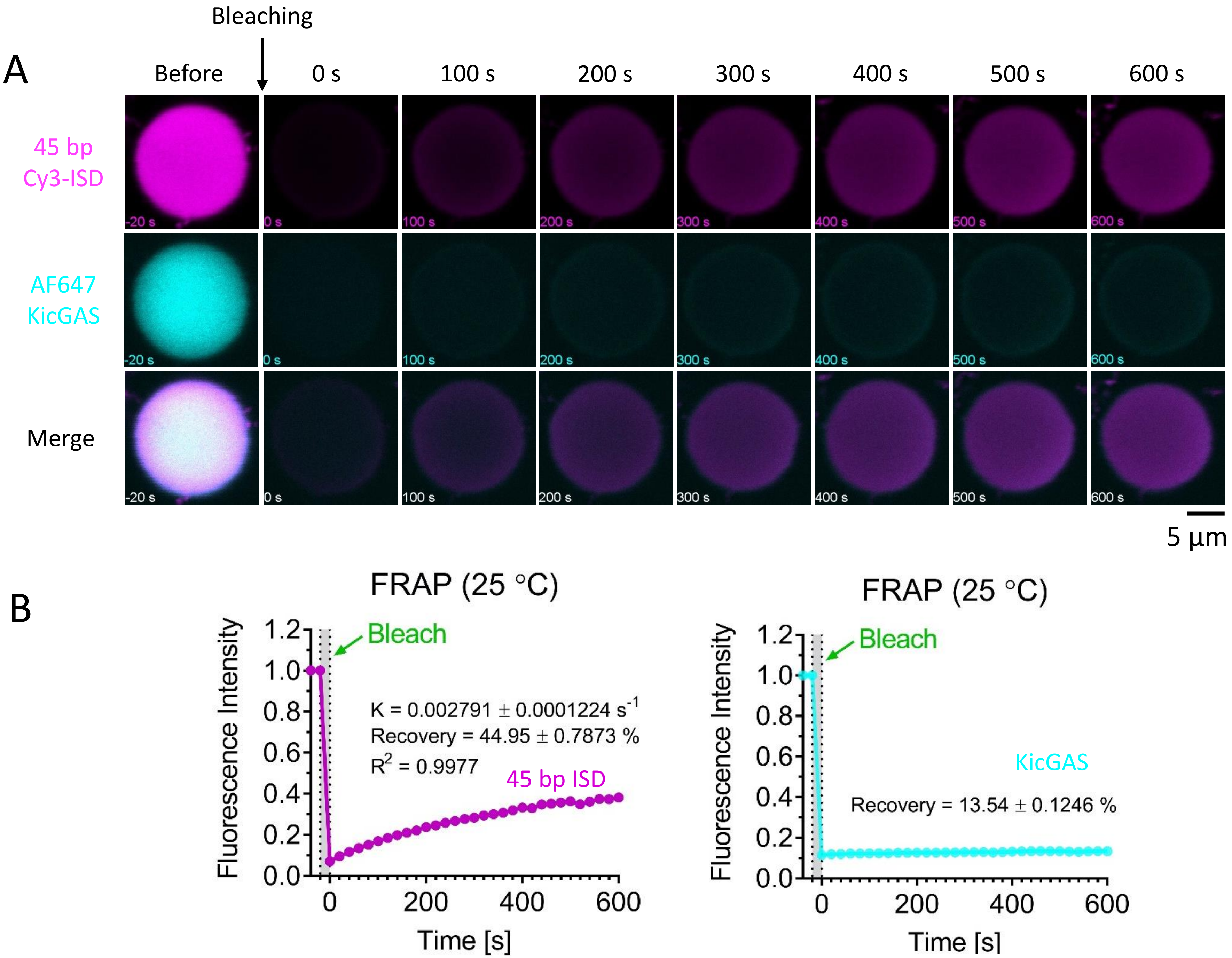

Figure S9. KicGAS and DNA molecules exchange less with molecules outside liquid droplets. (**A**) Representative micrographs of FRAP experiments on full bleaching of KicGAS–DNA condensates. (**B**) FRAP of KicGAS–DNA liquid droplets. Bleaching was performed at 30 min after KicGAS (30 µM) and DNA (30 µM) were mixed, and the recovery was allowed to occur at 25 ^{o}C. Time 0 indicates the start of recovery after photobleaching. n = 1 liquid droplet. AF647, Alexa Fluor 647.

Movie Legends

Movie S1. Representative time-lapse videos of FRAP experiments on partial bleaching of KicGAS–DNA condensates. (Corresponds to Fig. 6).

Movie S2. Representative time-lapse videos of FRAP experiments on full bleaching of KicGAS–DNA condensates. (Corresponds to Fig. S9).
