# Supplementary material for: Cooperative DNA binding mediated by KicGAS/ORF52 oligomerization allows inhibition of DNA-induced phase separation and activation of cGAS
Source: Nucleic Acids Res. 2021 Aug 13;49(16):9389–403. doi: 10.1093/nar/gkab689 (PMC8450086; doi:10.1093/nar/gkab689)
Supplement: gkab689_Supplemental_Files [file gkab689_supplemental_files.zip › Supplementary Movie legends.pdf]

Movie Legends

Movie S1. Representative time-lapse videos of FRAP experiments on partial bleaching of KicGAS–DNA condensates. (Corresponds to Fig. 6).

Movie S2. Representative time-lapse videos of FRAP experiments on full bleaching of KicGAS–DNA condensates. (Corresponds to Fig. S9).
